# Modulating task-outcome value to mitigate real-world procrastination via noninvasive brain stimulation

**DOI:** 10.7554/eLife.108241

**Published:** 2026-09-16

**Authors:** Zhiyi Chen, Zhilin Ren, Wei Li, Zhenzhen Huo, Zhuanzheng Wang, Ye Liu, Bowen Hu, Wanting Chen, Ting Xu, Leonov Artemiy, Chenyan Zhang, Bernhard Hommel, Tingyong Feng

**Affiliations:** 1 Faculty of Psychology, Southwest University Chongqing China; 2 Key Laboratory of Cognition and Personality, Ministry of Education Chongqing China; 3 Experimental Research Center for Medical and Psychological Sciences, School of Psychology, Third Military Medical University Chongqing China; 4 The Clinical Hospital of the Chengdu Brain Science Institute Chengdu China; 5 Key Laboratory for Neuroinformation, University of Electronic Science and Technology of China Chengdu China; 6 School of Psychology, Clark University Worcester United States; 7 Institute for Psychological Research, Leiden University Leiden Netherlands; University of California, Los Angeles United States; University of California, Los Angeles United States

**Keywords:** procrastination, noninvasive, neuromodulation, DLPFC, temporal decision model, Human

## Abstract

Procrastination is a prevalent behavioral problem associated with individual health and societal productivity. A leading model posits that procrastination reflects an imbalance between task aversiveness and the pursuit of positive task outcomes, yet this theoretical framework has neither been validated in real-world settings nor effectively applied to guide interventions. To address this gap, we conducted a double-blind, randomized, sham-controlled trial. Adults with chronic procrastination received seven sessions of high-definition transcranial direct current stimulation (HD-tDCS) to the left dorsolateral prefrontal cortex (DLPFC). Using the intensive experience sampling method, we assessed the effect of anodal HD-tDCS on real-world procrastination behavior at offline after-effect (2-day interval) and long-term after-effect (6-month follow-up). This neuromodulation produced a lasting reduction in real-world procrastination, with effects sustained at a 6-month follow-up. The mediation analysis indicated that increased outcome value, but not reduced task aversiveness, statistically accounted for variation in behavioral improvement. These findings are consistent with the hypothesis that enhancing DLPFC function may reduce procrastination by selectively amplifying the valuation of future rewards, not by reducing negative feelings about the task, which also suggests a targeted, theory-informed avenue for future behavioral interventions.

## Introduction

Procrastination is increasingly becoming a prevalent behavioral problem around the world, which reflects the irrational voluntary postponement of scheduled tasks albeit being worse off for such delays (Blake, 2019; Steel, 2007). In epidemiological investigations, more than 15% of adults were identified as having chronic procrastination problems, and the situation for students was worse as 70–80% of undergraduates engaged in procrastination (American College Health Association, 2022; Ferrari et al., 2005). Moreover, the behavioral genetic evidence indicates a certain heritability of procrastination in human beings as well (Gustavson et al., 2017; Gustavson et al., 2014; Gustavson et al., 2015). In addition to its prevalence, the undesirable associations between procrastination behavior and health also warrant caution. There is cumulative evidence to show the close associations between procrastination behavior and work performance, financial status, interpersonal relationships, and subjective well-being (Ferrari, 1994; Pychyl and Sirois, 2016; Steel et al., 2021). Further, as prospective cohort studies indicate, many mental health problems emerge alongside procrastination, particularly sleep problems, depression, and anxiety (Hairston and Shpitalni, 2016; Johansson et al., 2023). Even worse, chronic procrastination has been consistently associated with poor general health conditions, such as immune system disruption, gastrointestinal disturbance, hypertension, and cardiovascular disease (Sirois, 2015; Sirois, 2016). Thus, given these critical ramifications, considerable efforts have been devoted to delve into why we procrastinate irrationally.

To probe why we procrastinate irrationally, researchers have built upon theoretical bases of procrastination from different perspectives. For instance, Steel, 2007 pioneered a promising temporal motivation theory (TMT) to explicate procrastination as the failure of self-regulation. This theory suggests that individuals would procrastinate as task utility is devalued in the far future (Steel and König, 2006). Based on insights into emotional regulation, the mood repair perspective provides another explanation to elucidate that procrastination is due to the failure of self-regulation to give priority to short-term mood repair caused by doing the task rather than long-term task reward (Sirois and Pychyl, 2013). Recently, the temporal decision model (TDM) of procrastination further provides an integrative framework to explain the procrastination decisions, which highlights that procrastination is contingent on the trade-off between task aversiveness and task-outcome value (Zhang and Feng, 2020; Zhang et al., 2019b). Task aversiveness reflects how unpleasant individuals perceive tasks to be, with more unpleasant feelings making procrastination more likely (Zhang and Feng, 2020). Task-outcome value indicates how much it is worth as we evaluate the benefits it provides us (e.g., keeping body health) once we complete the task before the deadline (e.g., doing scheduled exercise) (Zhang and Feng, 2020). If the task aversiveness is overvalued in this trade-off, the decision to postpone tasks would be made consistently.

Theoretical frameworks posit that this trade-off and subsequent procrastination decisions may be modulated by individual differences in top-down regulatory capacity, with higher capacity typically associated with less procrastination (Blake, 2019; Saed, 2019; Zhao et al., 2021). Thus far, theoretical models have proposed three potential pathways through which regulatory processes might contribute to reduced procrastination: one for decreasing task aversiveness, the other one for increasing task-outcome value, and the last one for both (Zhang and Feng, 2020). As identified by both behavioral and neural evidence, procrastinators consistently report high task aversiveness when receiving scheduled tasks, and are more likely to postpone tasks so as to devalue negative task aversiveness (Blunt and Pychyl, 2000; Zhang et al., 2021). Meanwhile, prior literature suggests that top-down regulatory processes may facilitate the downstream modulation of negative emotional stimuli (Paschke et al., 2016; Tice and Bratslavsky, 2000). Therefore, one hypothesized pathway involves the downstream modulation of task aversiveness (Eckert et al., 2016). On the other hand, as a value-based decision, procrastination behavior is contingent on the evaluation of future outcomes (Rebetez et al., 2016; Zhang et al., 2019a). Existing evidence has shown that procrastinators generally underestimate the task-outcome value (Wu et al., 2016a; Zhang et al., 2021). In this vein, it is hard to generate the motivation to take immediate action (Taura et al., 2015). Notably, increasing the value of future rewards has been found effective in making individuals inclined to pursue future outcomes by potentially engaging prefrontal regulatory networks to prioritize future outcomes (Cho et al., 2015; Kelley et al., 2018). Thus, another pathway worth putting forward is that procrastination behavior could be shaped by increasing future task-outcome value. Also, given the integrative nature of prefrontal regulatory functions, we hypothesize a third pathway whereby both decreased task aversiveness and increased task-outcome value may jointly contribute to reduced procrastination, potentially reflecting coordinated downstream effects on valuation and affective processing.

Consistent with this framework, the left dorsolateral prefrontal cortex (DLPFC)—a region frequently implicated in value-based decision-making and top-down regulation—has been associated with procrastination. Structural and functional variations in the left DLPFC have been correlated with procrastination tendencies (Chen et al., 2020; Hu et al., 2018; Liu and Feng, 2017). In addition to structural brain hallmarks, the neurofunctional anomalies underlying procrastination were found in DLPFC-involved circuits (Wu et al., 2016b; Xu et al., 2021). Furthermore, the triple brain network theory provides network-based insights to highlight the neural signature of procrastination as the self-control neural network (e.g., left DLPFC; anterior cingulate cortex), emotional regulation network (e.g., insula and orbitofrontal cortex), and episodic prospection network (e.g., hippocampus and ventromedial prefrontal cortex, vmPFC, amygdala) (Chen and Feng, 2022; Chen et al., 2020; Schlüter et al., 2018; Wypych et al., 2019). In addition to the left lateralization, there is solid evidence indicating significant associations between top-down regulatory processes and the right DLPFC indeed, particularly given that this region specifically functions in top-down regulation, future self-continuity representation, and social decisions (Huang et al., 2025; Knoch and Fehr, 2007; Lin and Feng, 2024). Despite this case, Xu et al., 2023 demonstrated null effects of anodally stimulating the right DLPFC to modulate either value evaluation or emotional regulation for changing procrastination willingness. Moreover, a substantial amount of neural evidence supports this conclusion that DLPFC is involved in long-term reward evaluation and value encoding via top-down regulation circuits (Frost and McNaughton, 2017; Jimura et al., 2013; Smith et al., 2018). Using a neurocomputational model, Le Bouc and Pessiglione, 2022 provided clear evidence indicating that dorsal PFC signaling expected effort values was significantly attenuated in procrastinators compared to healthy controls (Le Bouc and Pessiglione, 2022). In this vein, this evidence supports this conceptualization that the left DLPFC may be a domain-specific neural signature determining one’s procrastination. In light of technical advances, high-definition transcranial direct current stimulation (HD-tDCS) has been widely used to reveal the potential neurocognitive mechanism of problematic behaviors by modulating cortical excitability, blood–brain barrier permeability, and even neuroplasticity (Cirillo et al., 2017; Woods et al., 2016), which is regulated by the *N*-methyl-D-aspartate system to either bolster LTP (long-term potentiation) or LTD (long-term depression) processes (Chrysikou et al., 2022; Shin et al., 2020). For instance, anodic HD-tDCS applied to the left DLPFC was found effective in inhibiting problematic behaviors caused by the lack of self-regulatory ability (Allenby et al., 2018), showing significantly amplified local neural oscillations (Chrysikou et al., 2022). Beyond regional neuromodulation in a dose-free protocol, cumulative evidence has well-documented that the effects of tDCS for neuromodulation are highly dose-dependent and are involved in network-wise covariance (Sabé et al., 2024; Soleimani et al., 2023; Woodham et al., 2025). Thus, this study aims to clarify the brain–behavior association between DLPFC neuromodulation and procrastination, and to test whether observed changes in task valuation and aversiveness are consistent with theoretical models of top-down regulation.

**Figure 1.**
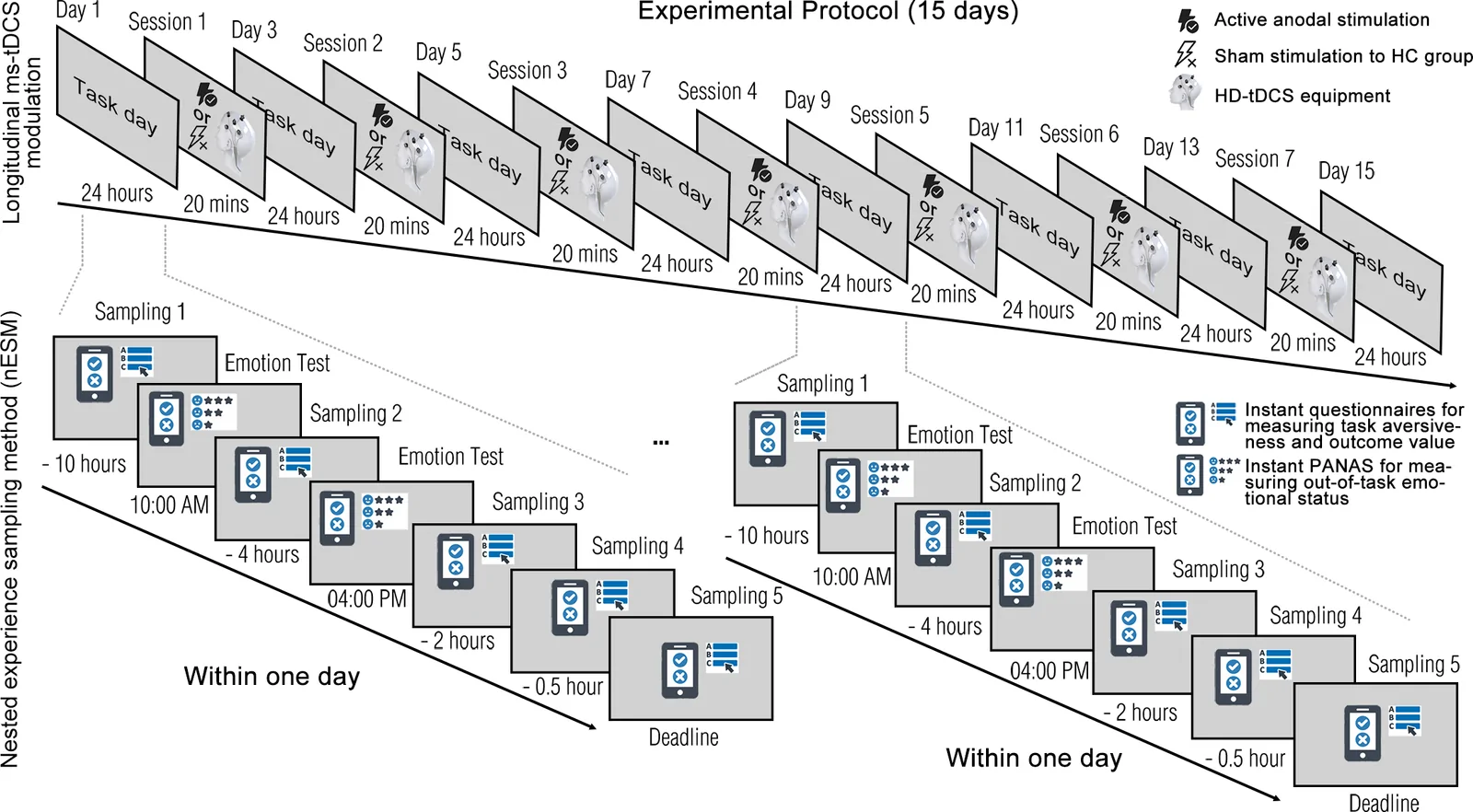
Experimental diagram of this study. The upper sub-graph illustrates the whole multiple sessions tDCS neuromodulation pipeline, including seven sessions (days) and eight task-demanded days. Here, the ‘flash’ icon indicates conducting tDCS neuromodulation (active anodal stimulation for active NM group and sham stimulation for sham NM group). This ‘+’ label means a task-demanded day, where no stimulation is required for participants, but all the covariates of interest should be measured by experience sampling methods. The bottom sub-graph reflects the specific pipeline in task-demanded days. Participants were required to provide responses at five progressive time moments nearing the deadline for task aversiveness and outcome value in task-demanded days. In this diagram, the icon of ‘clock’ symbolizes ecological momentary assessment for measuring instant task willingness and outcome value. In addition, twice tests for daily emotions (labeled by ‘+’) were added for participants at 10:00 and 16:00 as covariates of no interests to be adjusted.

**Figure 2.**
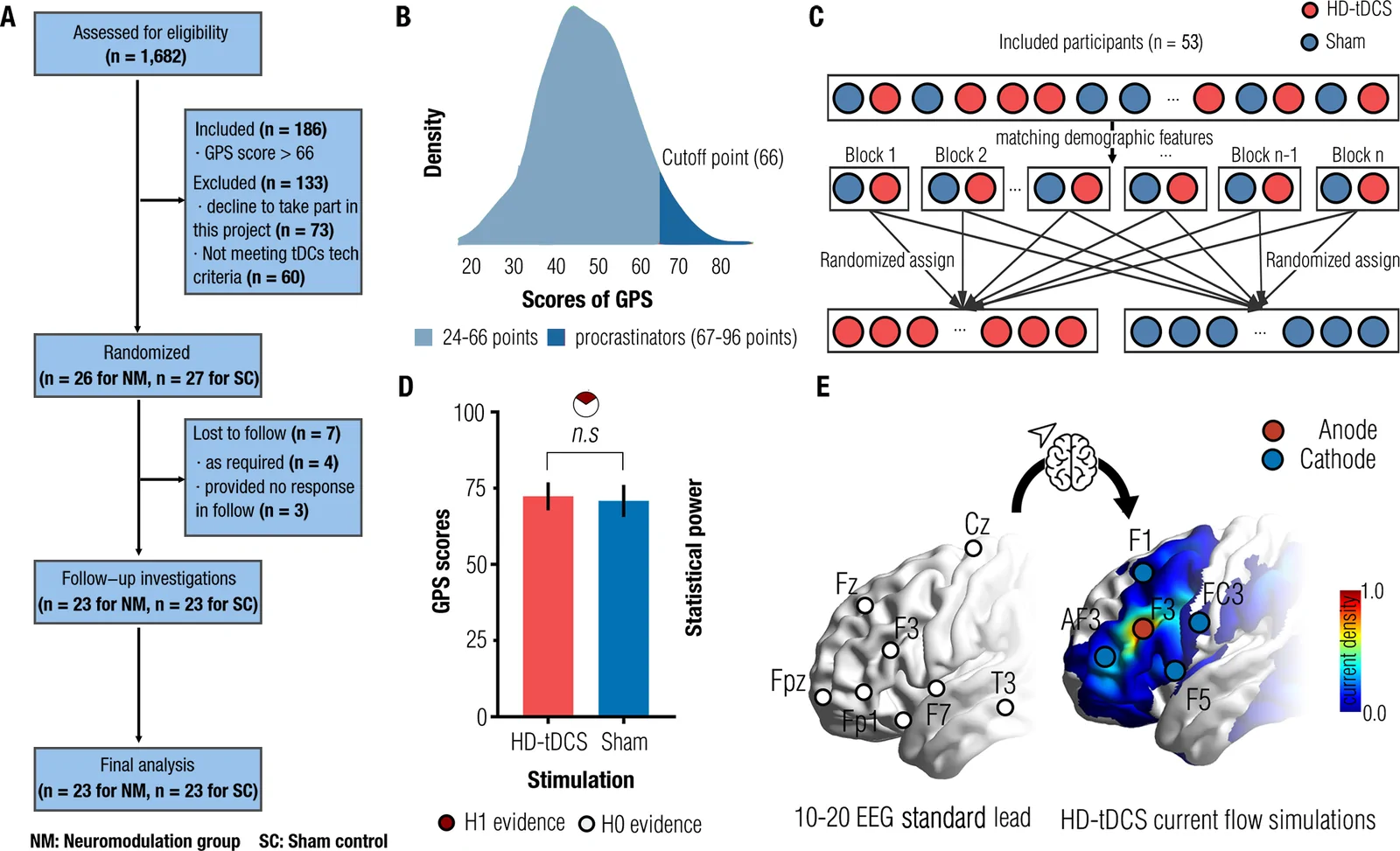
Flow diagram of CONSORT (**A**) and partial details of randomized groups (**B–D**) and neural locations of electric pole (**E**). (**B**) plots the distribution of all the participants' procrastination scores (GPS = General Procrastination Scale). (**C**) detailed what the full randomized block design is. (**D**) shows the comparison between the active neuromodulation group and sham control for procrastination scores. Each bar indicates the mean value, and the error bars reflect the standard deviation (SD). n.s. indicates no statistically significant difference. (**E**) indicates the pipeline to determine the location of the electric pole. The 10–20 EEG standard lead is used to locate the left dorsolateral prefrontal cortex (DLPFC) initially, and the neuronavigation is further utilized to locate the exact location of this targeting region (i.e., left DLPFC).

To empirically evaluate these theoretical pathways, we conducted a double-blind, randomized, multiple-session, sham-controlled design, with a 2 (active HD-tDCS vs. sham control) × 2 (before first neural stimulation vs. after last neural stimulation) full factorial design (see Figures 1 and 2). Participant demographic characteristics and baseline measures are summarized in Table 1. This HD-tDCS protocol consisted of seven sessions spaced over 15 days, and each session was implemented every 2 days. To ensure sound ecological validity, each procrastinator was informed to report one REAL-LIFE task that he/she should complete the following day (e.g., Day 2) after the current neuromodulation session (e.g., Day 1), and was asked to report the ACTUAL performance for completing this task in that day (Day 2) (see Figure 1). Based on the TDM of procrastination (Zhang et al., 2019b), we drew on the experience sampling method (ESM) to estimate the real dynamics of task aversiveness and task-outcome value by using a parameter-free model in each session. More importantly, to clarify which pathway best explains the neurocognitive mechanisms of procrastination, we built upon the mixed-effects general linear model and Quasi-Bayesian mediation model to test whether the changes in task aversiveness and task-outcome value modulated by HD-tDCS could predict decreased procrastination. Finally, the follow-up investigation for actual procrastination was conducted for 6 months after the experiment to examine the long-term after-effect of this neural stimulation.

**Figure 2—figure supplement 1.**
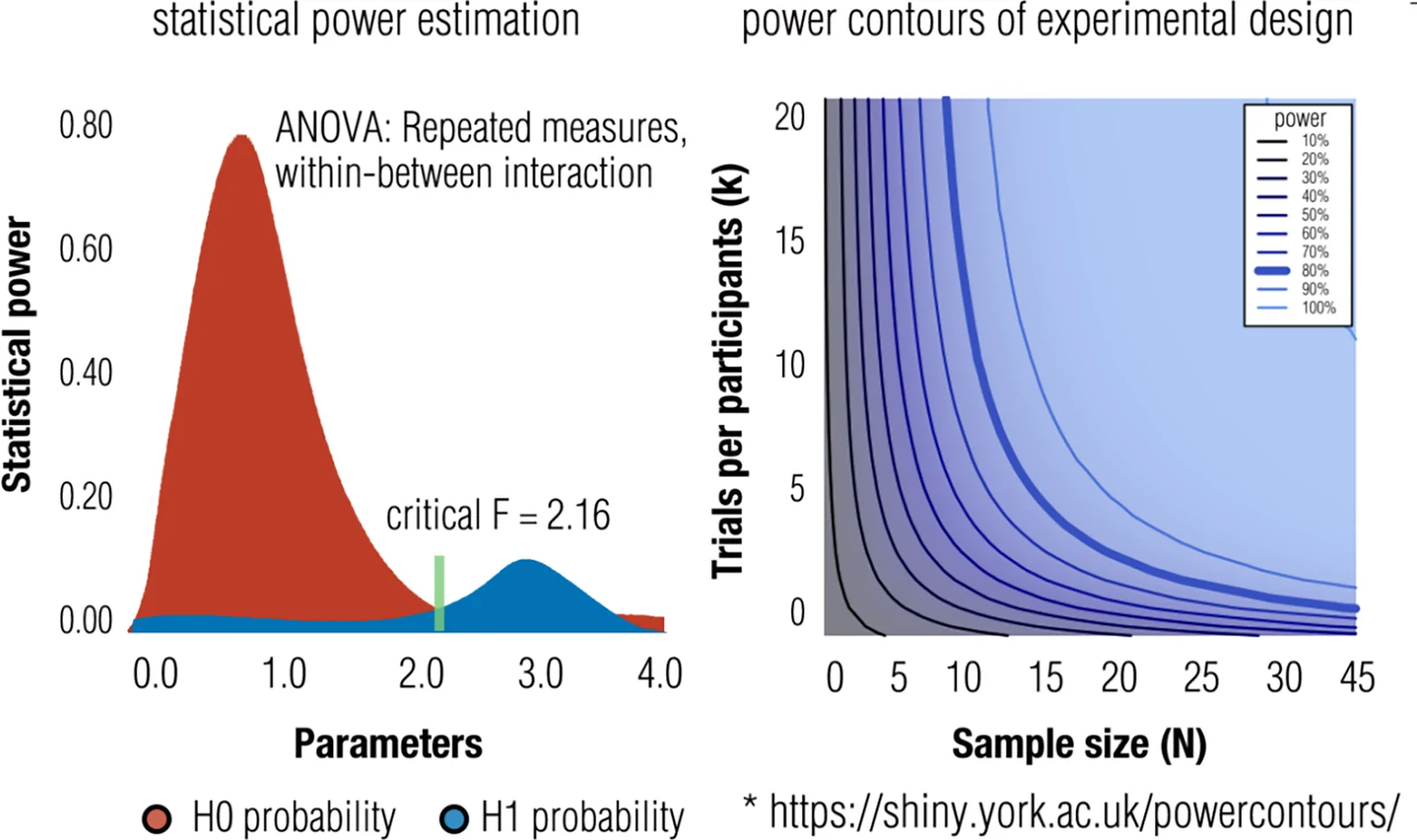
Statistical power estimation by using ANOVA model and power contours according to experimental design.

**Table 1.** Demographic information for included participants. NM represents active neuromodulation group and sham indicates sham-control group. Anxiety symptoms were measured by State-Trait Anxiety Inventory (STAI). Depression symptoms were tested by Self-Rating Depression Scale (SDS). BF10 describes the Bayesian evidence strength to support the alternative hypothesis, with >3 for strong evidence.

	Active NM		SC		p value (BF_10_)
	Male	Female	Male	Female	
Gender	3	20	3	20	0.99 (-)
Age	19.61 ± 0.78		22.22 ± 1.44		0.08 (1.03)
SES	2.17 ± 0.65		2.34 ± 0.64		0.38 (0.41)
Anxiety	48.48 ± 6.52		47.30 ± 6.87		0.56 (0.33)
Depression	47.13 ± 8.27		47.50 ± 9.28		0.88 (0.29)
Procrastination	71.00 ± 5.47		72.07 ± 4.77		0.26 (0.48)

## Results

### Blinding

In both groups, almost all participants reported perceiving acceptable pain stemming from current stimulation and believed they were receiving treatment, with 91.30% (21/23) for the active neuromodulation group (NM) and with 86.95% (20/23) for the sham-control group (SC) (*x*^2^ = 0.224, p = 0.636). All the participants were engaged in the identical experimental procedures, excepting stimulation’s type (active vs. sham). In addition, statistical models excluded sessions 1 and 4 because participants reported additional unexpected events that uncontrollably disrupt task execution in both groups (see Appendix 1 Results and Appendix 1—table 2).

### Multiple-session HD-tDCS can alleviate procrastination

**Figure 3.**
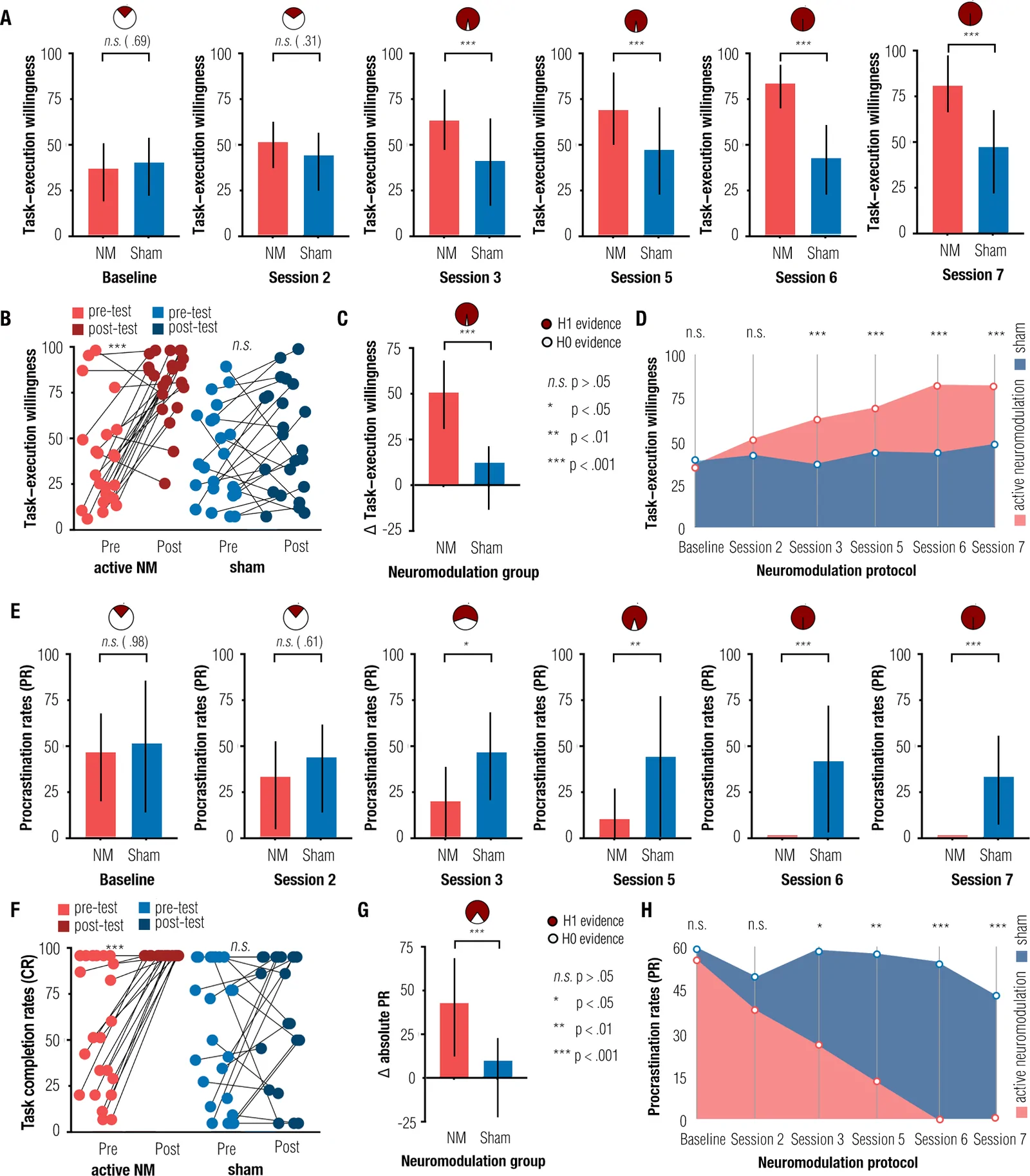
Results of neuromodulation effects to task-execution willingness and procrastination rates (PRs). (**A**) shows the effects of neuromodulation to increase task-execution willingness for both active group and sham control across sessions that included in formal analysis (sessions 0 (baseline), 2, 3, 5, 6, and 7). (**B**) illustrates the effects of whole neuromodulation round to task-execution willingness for both groups. (**C**) plots the changes of task-execution willingness for both groups after neuromodulation. (**D**) provides a line chart to show the changes of task-execution willingness across each session that included in formal analysis. (**E**) shows the effects of neuromodulation to reduce PR for both active group and sham control across sessions that included in formal analysis. (**F**) illustrates the effects of whole neuromodulation round to task-completion rate for both groups. (**G**) plots the absolute changes of PR for both groups after neuromodulation. (**H**) provides a line chart to show the changes of PR across these sessions that are included in formal analysis. The pie graph for each comparison represents the corresponding result of Bayesian factor inference, with the brown piece for supporting H1 evidence and the white piece for supporting H0 evidence. Each bar indicates the mean value, and each line placed onto the bar reflects the standard deviation (SD). Each group included 23 participants. Individual pre- and post-neuromodulation observations are shown in panels B and F, with lines connecting observations from the same participant. Group-by-session effects were examined using linear mixed-effects models, and pre–post simple effects were evaluated using Tukey-adjusted comparisons of estimated marginal means. Trends across sessions were assessed using the Mann–Kendall test.

To identify whether multiple-session HD-tDCS (ms-tDCS) targeting the left DLPFC can alleviate subjective procrastination willingness and actual procrastination behavior, a general mixed-effects linear model (LMM) with Satterthwaite’s method was built, with task-execution willingness (TEW) and actual procrastination rates (PR) as primary outcomes, respectively. For procrastination willingness, results showed a statistically significant interaction effect between multi-session neuromodulations and groups (*β* = –7.84, SE = 1.80, *t* = –4.36, DF = 45.6, p < 0.001, Cohen *d* = 2.37, 95% CI: [1.49–3.25]; Figure 3A, Figure 3—figure supplement 1a). In the post hoc simple effect analysis, it demonstrated a significantly increased TEW (i.e., decreased procrastination willingness) after neuromodulation in the active neuromodulation group (NM-before: 35.65 ± 30.21, NM-after: 80.43 ± 19.92, Mean Diff = 41.79, SE = 7.58, DF = 103.4, *t*.ratio = 5.51, p < 0.0001, Tukey correction), but no such effects were identified in the sham-control group (SC-before: 37.57 ± 26.46, SC-after: 47.35 ± 30.49, Mean Diff = 2.58, SE = 7.56, DF = 96.8, *t*.ratio = 0.34, p = 0.73, Tukey correction) (Figure 3B, C). A linear uptrend for TEW was further observed across multiple sessions in the active NM group, indicating gradually increasing neuromodulation effects (Figure 3D; p < 0.01, Mann–Kendall test). For actual procrastination behavior, changes to actual PRs across all the sessions have been detailed in Figure 3E, Figure 3—figure supplement 1b. Similarly, a statistically significant interaction effect was identified here (*β* = –7.37, SE = 2.40, *t* = –3.02, DF = 46.6, p = 0.004, Cohen *d* = 1.52, 95% CI: [0.87–2.16]), and the simple effect analysis further revealed decreased actual PRs after ms-tDCS in the active neuromodulation group (NM-before: 43.26 ± 39.10, NM-after: 0.00 ± 0.00, Mean Diff = 44.40, SE = 9.36, DF = 110.0, *t*.ratio = 4.74, p < 0.0001, Tukey correction), but no such prominent changes were found in the sham-control group (SC-before: 46.48 ± 40.76, SC-after: 33.35 ± 37.82, Mean Diff = 7.53, SE = 9.28, DF = 102.0, *t*.ratio = 0.81, p = 0.42, Tukey correction) (Figure 3F, G). Also, a significant downtrend for PRs across all the sessions was identified in the active NM group (Figure 3H; p < 0.01, Mann–Kendall test).

**Figure 3—figure supplement 1.**
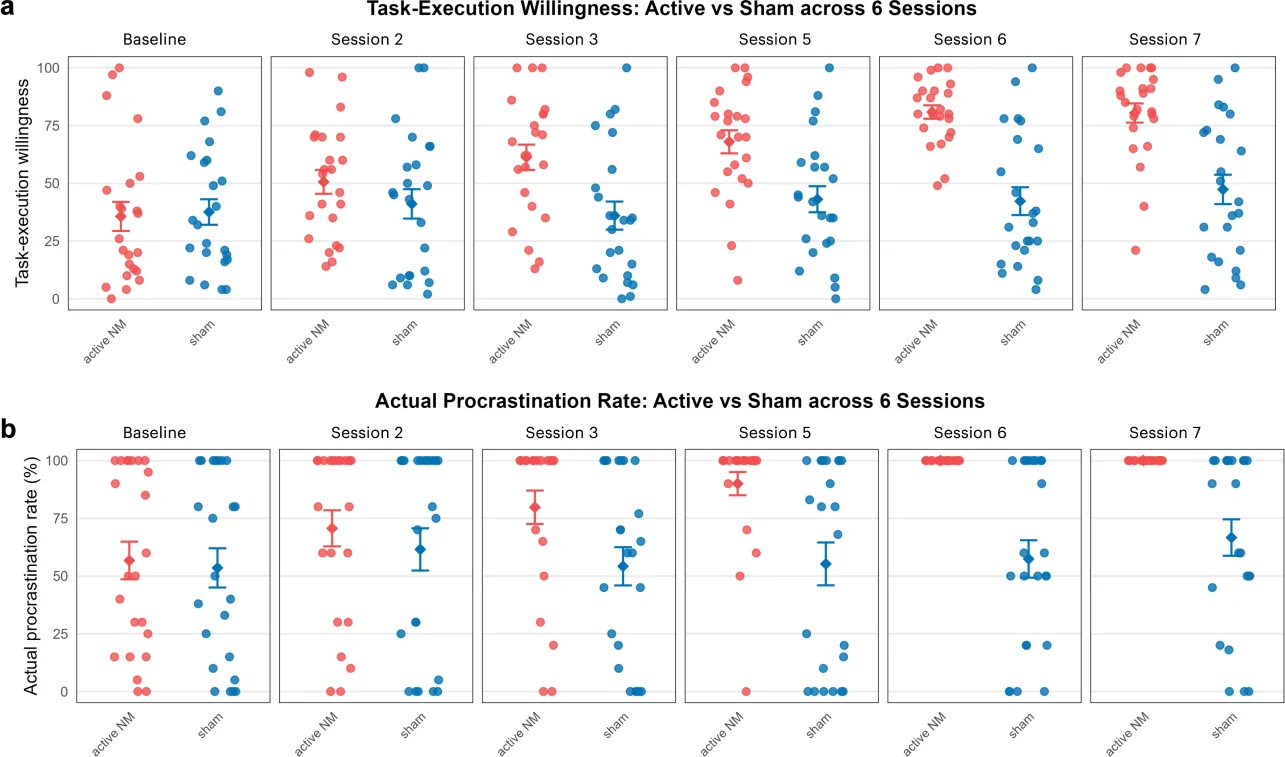
Jittered dot points for primary outcomes in the present study.

Furthermore, the nonparametric *x*^2^ test of *R* × C contingency table was conducted for the count of procrastinated tasks, by treating this outcome (i.e., whether a participant actually procrastinates the task in real-world settings) as an ordinal variable. Results showed a significant reduction in group-averaged procrastination frequency in the active neuromodulation group after the last-session HD-tDCS, but not in the sham-control group (NM-before: 69.56% (16/23 participants), NM-after: 0.00% (0/23 participants); SC-before: 69.56% (16/23 participants), SC-after: 56.52% (13/23 participants), *x*^2^ = 10.08, p < 0.001), partly indicating a high statistical robustness. To systematically test whether such effects are biased by extreme data points or patterns, we reran the main LMMs by iteratively removing data from the last two sessions, which showed extraordinarily high effectiveness from neuromodulation (e.g., all the participants in the neuromodulation group had no actual procrastination behavior in sessions #6 and #7). Results showed the significant group*neuromodulation sessions interaction effects across all those nested models (removing sessions #6, #7, or both, all p < 0.05; see Appendix 1 Results and Appendix 1—Tables 3 and 4), potentially indicating a statistical robustness from the data pattern. Furthermore, as a sensitivity analysis, we reran the main LMMs using Beta regression distribution with a logit link function, which is appropriate for both bounded outcomes mentioned above (i.e., procrastination rate and procrastination willingness). The main effects (i.e., Group and Treatment day) and their interaction remained significant for both procrastination rate and willingness (see Appendix 1 Results and Appendix 1—table 5), confirming that our findings are robust to alternative distributional assumptions. In brief, these findings provided empirical evidence to support that ms-tDCS neuromodulation targeting the left DLPFC can be an effective way to reduce both procrastination willingness and actual procrastination.

### Ms-tDCS changes task aversiveness and task-outcome value

**Figure 4.**
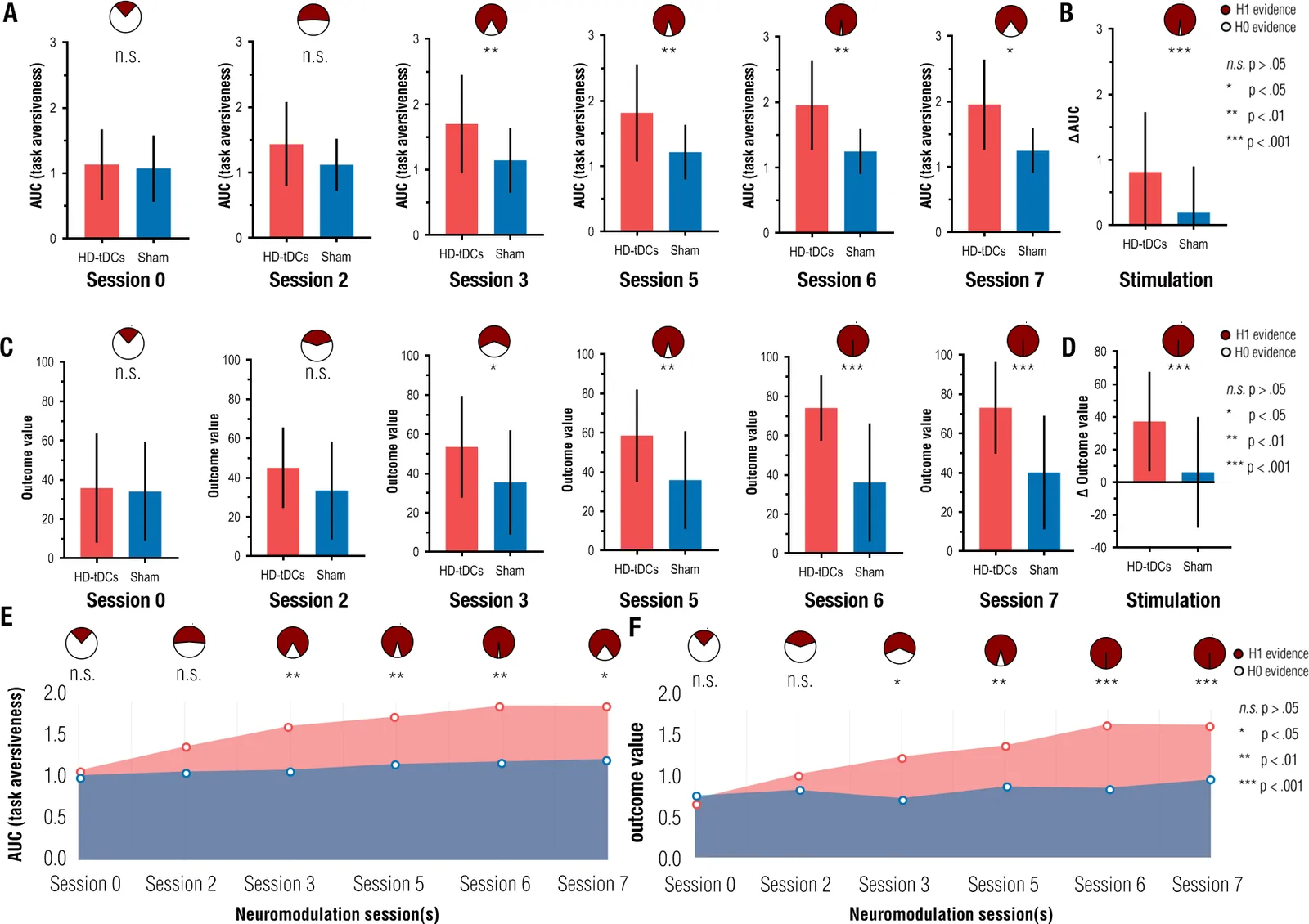
Results of neuromodulation effects to task aversiveness and outcome value. (**A**) shows the effects of neuromodulation to increase AUC of task aversiveness for both active group and sham control across sessions that included in formal analysis (sessions 0 (baseline), 2, 3, 5, 6, and 7). Higher AUC indicates lower task aversiveness as a given task is increasingly executed. (**B**) plots the changes of AUC of task aversiveness for both groups after neuromodulation. (**C**) shows the effects of neuromodulation to increase outcome value for both active group and sham control across sessions that included in formal analysis. (**D**) plots the changes of outcome value for both groups after neuromodulation. (**E**) provides a line chart to show the changes of AUC of task aversiveness across these sessions that included in formal analysis. (**F**) provides a line chart to show the changes of outcome value in the same manner. Pie graph for each comparison represents the corresponding result of Bayesian factor inference, with brown piece for supporting H1 evidence and white piece for supporting H0 evidence. Each bar indicates mean value, and each line placed onto the bar reflects standard deviation (SD). Each group included 23 participants. Group-by-session effects were examined using linear mixed-effects models, and pre–post simple effects were evaluated using Tukey-adjusted comparisons of estimated marginal means. Trends across sessions were assessed using the Mann–Kendall test.

Both task aversiveness and task-outcome value serve as key pathways determining whether one would procrastinate. To this end, we further utilized a generalized linear mixed-effects model to examine the effects of ms-tDCS on changes in task aversiveness and task-outcome value. Task aversiveness changes across all the sessions are shown in Figure 4A–C, Figure 4—figure supplement 1. We demonstrated a statistically significant decrease in task aversiveness and an increase in task-outcome value via ms-tDCS in the neuromodulation group (Task aversiveness: interaction effect, *β* = –0.12, SE = 0.04, DF = 46.69, *t* = –3.28, p = 0.002; simple effect, NM-before_(AUC)_: 1.13 ± 0.54, NM-after_(AUC)_: 1.95 ± 0.84, Mean Diff = 0.81, SE = 0.16, DF = 104.5, *t*.ratio = 4.91, p < 0.001, Tukey correction; Outcome value: *β* = –6.76, SE = 1.74, DF = 46.2, *t* = –3.89, p < 0.001; simple effect, NM-before: 35.87 ± 27.83, NM-after: 73.09 ± 23.34, Mean Diff = 39.36, SE = 7.25, DF = 103.7, *t*.ratio = 5.43, p < 0.001, Tukey correction; see Figure 4B), but not in the sham-control group (Task aversiveness: SC-before_(AUC)_: 1.07 ± 0.51, SC-after_(AUC)_: 1.28 ± 0.46, Mean Diff = 0.17, SE = 0.17, DF = 98.3, *t*.ratio = 1.06, p = 0.29, Tukey correction; Outcome value: SC-before: 34.00 ± 25.17, SC-after: 40.13 ± 28.94, Mean Diff = 5.54, SE = 7.26, DF = 98.7, *t*.ratio = 0.76, p = 0.44, Tukey correction; see Figure 4D). In the neuromodulation (NM) group, task aversiveness steadily decreased with the cumulative number of stimulation sessions, while perceived task-outcome value increased significantly (see Figure 4E, F, p < 0.05, Mann–Kendall test). Thus, these findings are consistent with the view that neuromodulation of the left DLPFC is associated with reduced task aversiveness and increased task-outcome value.

**Figure 4—figure supplement 1.**
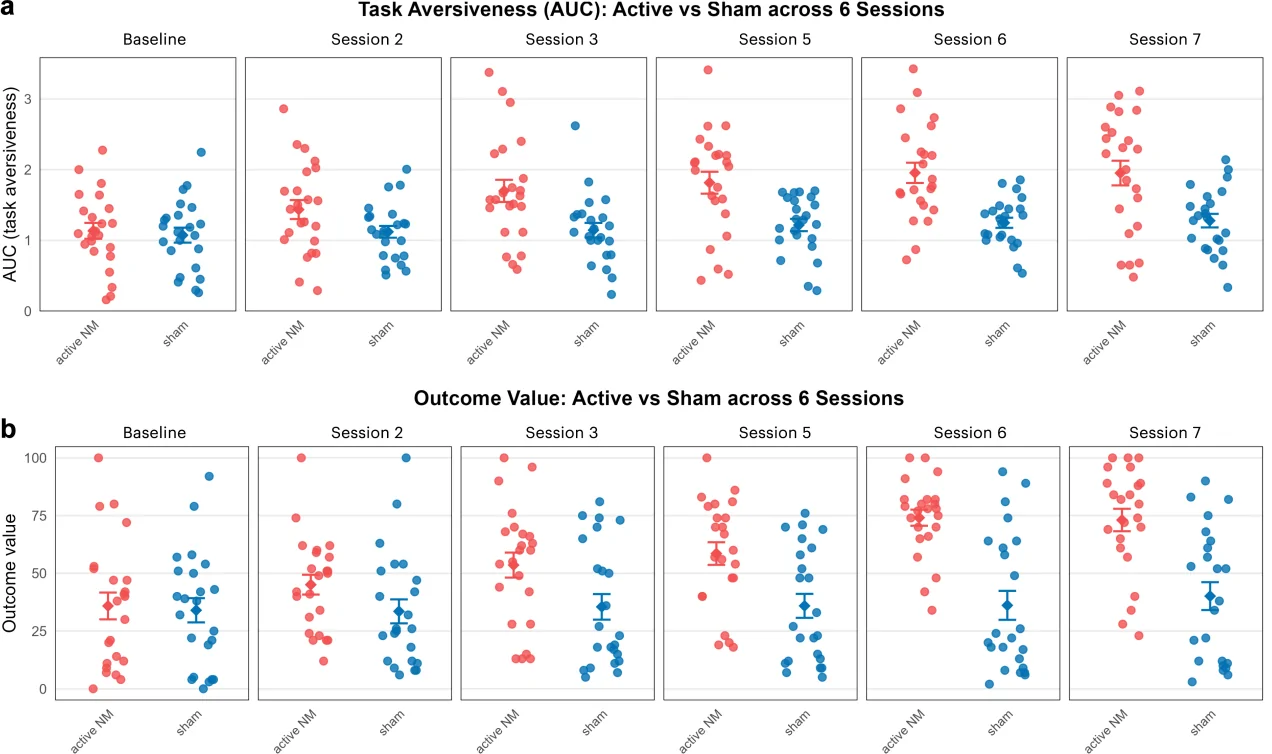
Jittered dot points for secondary outcomes in the present study.

### Increased task-outcome value but not decreased task aversiveness predicts reduced procrastination

Given the dual neurocognitive pathways identified above—reduced task aversiveness and increased task-outcome value—we proposed that these changes may reflect downstream consequences of prefrontal neuromodulation on value-based decision processes, statistically consistent with theoretical models of top-down regulation. To this end, we utilized a general linear model to regress decreased task aversiveness and increased task-outcome value to changes in TEW. In this model, increased task-outcome value (Δ_Outcome value_) significantly predicted increased TEW (Δ_task-execution willingness_) (*x*^2^ = 15.95, p < 0.01, *R*^2^ = 0.40; Δ_Outcome value_: *β* = 0.61, SE = 0 .12, p < 0.001, 95% CI: [0.37–0.86]), whereas no significant effect was observed for predicting TEW through decreased task aversiveness (Δ_Task aversiveness_: *β* = 0.10, SE = 0.12, p = 0.41, 95% CI: [−0.14 to 0.34]). Likewise, for actual procrastination behavior in real-world settings, increased outcome value (Δ_Outcome value_) was identified to be significantly predictive, whereas decreased task aversiveness showed null effects (see Table 2). Collectively, these findings provide statistical evidence consistent with the hypothesis that the outcome-value pathway may contribute to procrastination reduction.

**Table 2.** Summary for general linear model in predicting changes of task aversiveness and outcome value to actual procrastination. S.E. means standard error. *p < 0.05.

	*β*	SE	p value	Odd ratio (OR)	adj *R*^2^
Δ_Task aversiveness_	0.62	0.42	0.13	1.85	0.29
Δ_Outcome value_	0.85*	0.42	0.04	2.34	

### Increased task-outcome value is specifically associated with reduced procrastination in the contexts of neuromodulation

**Figure 5.**
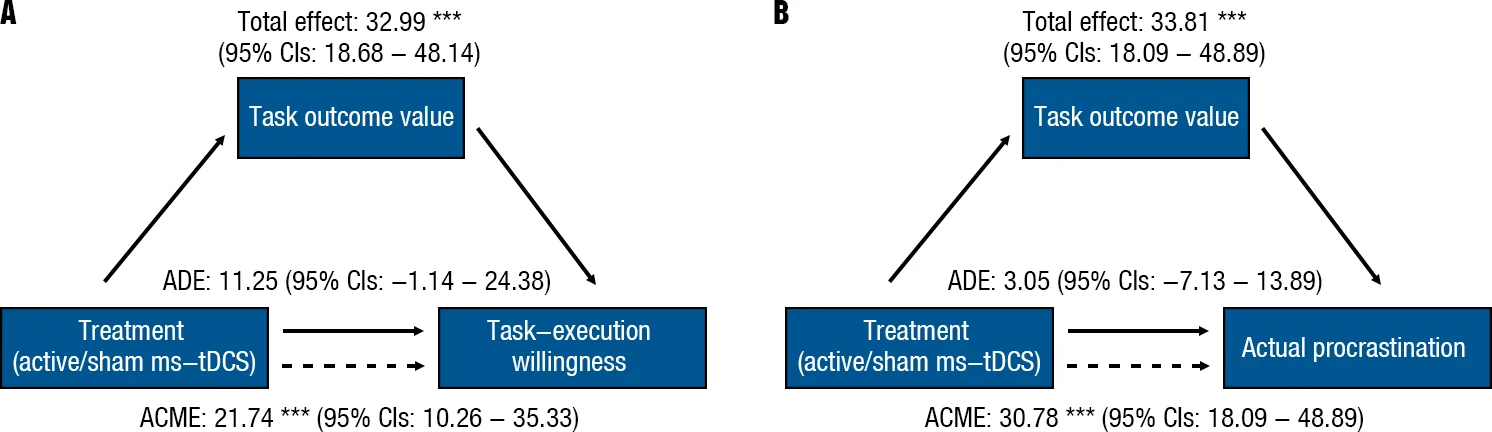
Results of Quasi-Bayesian model for the mediated role of increased task-outcome value in the association between neuromodulation and task-execution willingness (**A**) and actual procrastination rates (**B**). ADE = average direct effect, ACME = average causal mediation effect, CI = confidence interval, ***p < 0.001.

To explore the potential neurocognitive pathways of procrastination, the Quasi-Bayesian mediation analysis was undertaken, with increased task-outcome value as a statistically mediated variable. As an exploratory analysis, results indicated that increased task-outcome value was associated with changes in the TEW (*δ* = 21.74, p < 0.01; *ζ* = 11.25, p = 0.07, *ρ* = 32.99, p < 0.01, simulation = 1000; see Figure 5A) and real-world procrastination (*δ* = 30.78, p < 0.01; *ζ* = 3.05, p = 0.52, *ρ* = 33.81, p < 0.01, simulation = 1000; see Figure 5B), in the context of ms-tDCS neuromodulation. To ensure the statistical robustness and specificity of these findings, the sensitivity analysis was implemented by changing sampling parameters and outcome variables. By doing so, those findings were validated statistically robust, as shown by replicated observations across bootstrapping sampling subsets (see Appendix 1 Results and Appendix 1—Tables 6 and 7). Moreover, the results of the control analysis further validated the specificity of these findings by showing a null statistically mediated effect of this model to predict one’s task aversiveness (see Appendix 1 Results and Appendix 1—table 8). In summary, these findings identified a statistical pathway consistent with the theoretical model: neuromodulation of the left DLPFC was associated with increased task-outcome value, which in turn was associated with reduced procrastination. Nevertheless, all mediation findings are now explicitly labeled as ‘exploratory’ and framed as quantifying statistical associations consistent with the TDM pathway, not causal mediation.

### Long-term effects of ms-tDCS

**Figure 6.**
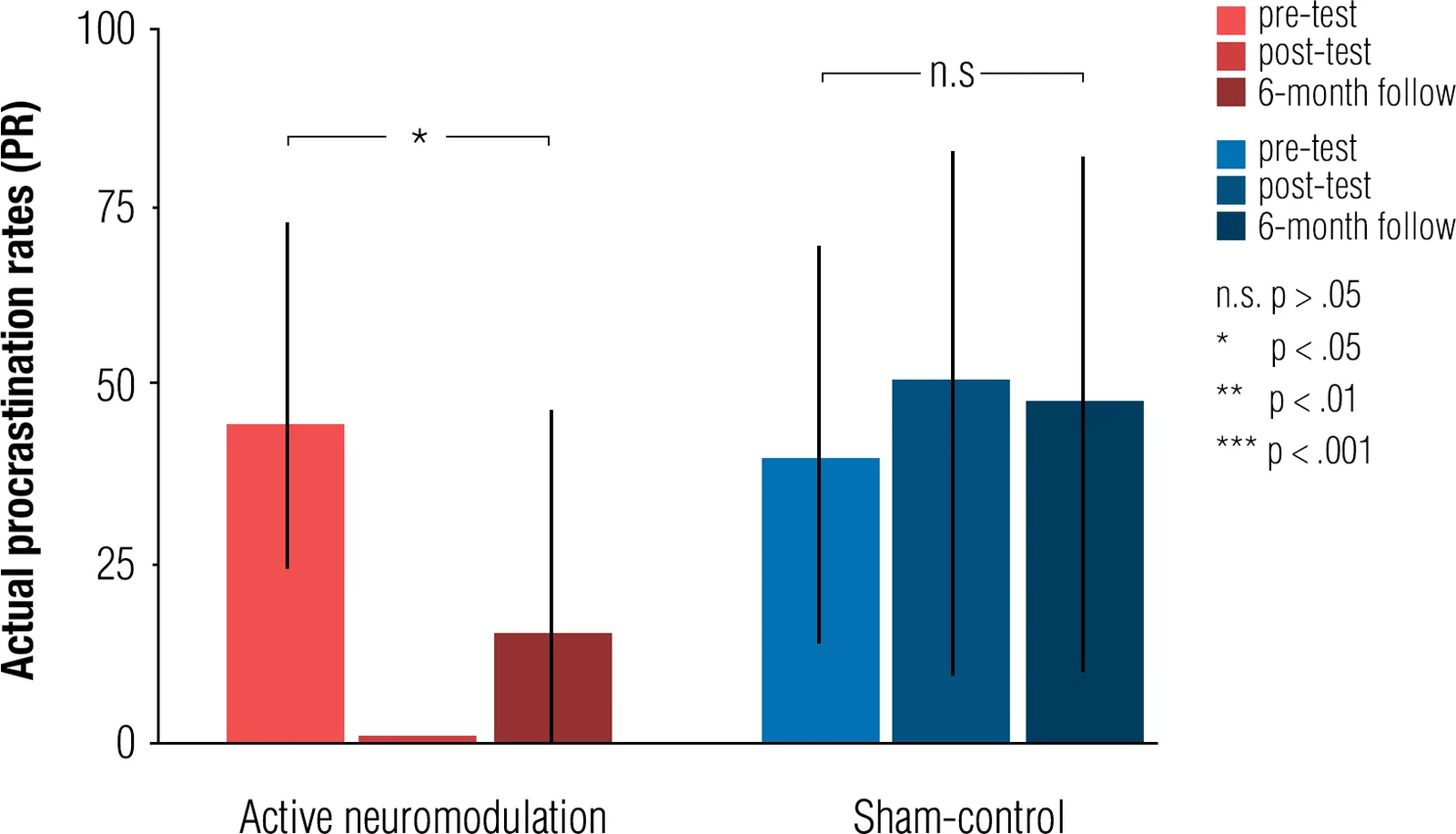
Changes of actual procrastination rates among pre-test, post-test, and 6-month follow-up for active neuromodulation group and sham-control group. Pre-test means to test the actual procrastination rates before first HD-tDCS neuromodulation. Post-test means to test the actual procrastination rates after last neuromodulation. The 6-month follow-up means to re-investigate the actual procrastination rates after last neuromodulation for 6 months. Each bar indicates the mean value, and the error bars reflect the standard deviation (SD). At the 6-month follow-up, 22 participants in the active neuromodulation group and 23 participants in the sham-control group were assessed. Baseline-to-follow-up comparisons were evaluated using a linear mixed-effects model with Tukey-adjusted simple-effect comparisons. Brackets indicate comparisons between pre-test and the 6-month follow-up within each group.

We have also attempted to conduct a follow-up investigation to test the long-term after-effect of ms-tDCS in reducing actual procrastination. Almost all the participants had undergone follow-up except one in the neuromodulation group after last neuromodulation for 6 months (*N*_NM_ = 22, *N*_SC_ = 23). Thus, the LMM was constructed, with the PR before first neuromodulation vs. PR after last neuromodulation for 6 months as covariates of interest. Results showed the statistically significant group*time interaction effects (*β* = 16.5, SE = 9.9, p = 0.049). Simple-effect model demonstrated a decrease in actual PRs in the active neuromodulation group after last stimulation for 6 months compared to baseline (*β* = –22.05, SE = 10.0, p = 0.038, Tukey correction; NM-before: 40.68 ± 37.96, NM-after_6-months_: 18.63 ± 29.80), and revealed null effects in the SC group (*β* = 1.26, SE = 9.78, p = 0.99, Tukey correction; SC-before: 46.47 ± 40.75, SC-after_6-months_: 47.73 ± 39.18) (see Figure 6). Furthermore, using a nonparametric *x*^2^ test to compare differences in the number of procrastinated tasks, we still found a statistically significant reduction in procrastination frequency in NM group after neuromodulation for 6 months compared to baseline (*x*^2^ = 3.30, p = 0.035, NM-before: 68.19% (15/22), NM-after_6-months_: 40.91% (9/22)), while no significant changes were observed in the SC group (*x*^2^ = 0.11, p = 0.74, SC-before: 69.56% (16/23), SC-after_6-months_: 73.91% (17/23)). Therefore, beyond short-term effects, the benefits of ms-tDCS neuromodulation on reducing procrastination were still detectable at a 6-month follow-up, providing preliminary evidence consistent with long-term after-effects.

## Discussion

In the current study, by performing anodal ms-tDCS neuromodulation on the left DLPFC, both procrastination willingness and actual procrastination behavior were significantly decreased in real life. Additionally, a 6-month follow-up investigation revealed the long-term retention of such effects. Furthermore, this neuromodulation was found to decrease task aversiveness and increase outcome values; notably, only increased task-outcome value could predict decreased procrastination. On balance, our findings provide evidence consistent with the hypothesis that neuromodulation of the left DLPFC is associated with reduced procrastination, primarily through increasing task-outcome value rather than merely reducing task aversiveness. In addition, this study provided an effective way to reduce actual procrastination by using ms-tDCS neuromodulation.

One contribution of this study is to provide empirical evidence partially consistent with the TDM, showing that increased task-outcome value—rather than decreased task aversiveness—was statistically associated with reduced procrastination following DLPFC neuromodulation. Neurobiological substrates of procrastination have been investigated in recent years, and have demonstrated the crucial roles of the left DLPFC in predicting procrastination (Chen and Feng, 2022; Chen et al., 2021; Chen et al., 2022; Hu et al., 2018; Liu and Feng, 2017; Zhang et al., 2017; Zhang et al., 2016). Not only the brain functional anomalies of the left DLPFC but also the neuroanatomical disruptions of self-regulatory brain network constituted by the left DLPFC were found to be linked with more procrastination behaviors (Chen and Feng, 2022; Zhang et al., 2016). Notwithstanding this, it still remains unclear to claim their brain-behavior relationship—that is—no known evidence existed to clarify whether changes of the left DLPFC lead to procrastination or vice versa. The current study demonstrated the role of the left DLPFC in procrastination by showing that the neuromodulation of the left DLPFC indeed manipulated procrastination, and thus provided straightforward and powerful evidence to fill this gap.

It has long been acknowledged that the left DLPFC is consistently implicated in top-down regulatory processes and value-based decision-making, such as patience to wait long-term gratification for a delay, inhibition of impulsiveness, and control of game addiction (Cohen and Lieberman, 2010; Lin and Feng, 2024). Furthermore, the increased activation of the left DLPFC has been observed during the exertion of self-regulation which modulates value signals (Hare et al., 2009; Harris et al., 2013). Meanwhile, procrastination has been argued to be the consequence of self-regulation failure for a long time (Ariely and Wertenbroch, 2002; Rebetez et al., 2018; Rozental and Carlbring, 2014). Supporting this, both the brain morphological disruptions in the DLPFC and anomalies in the functional coupling of the DLPFC-based regulatory network have been correlated with procrastination tendencies (Xu et al., 2021; Yang et al., 2021). Moreover, it was worth noting that the increased activation of the left DLPFC was found to be involved in outcome value evaluation through self-control regulation (Chen et al., 2018; Zha et al., 2019). There is more straightforward evidence to substantiate the role of manipulating the DLPFC in changing one’s subjective value evaluation (Huang et al., 2017; Minati et al., 2012). Moreover, the theoretical explanations and empirical evidence have increasingly converged into one line for claiming that the increased task-outcome value would prompt more motivation to drive one to take action immediately and thus reduce procrastination (Zhang et al., 2019b). Building on this foundation, among several theoretical interpretations and cognitive pathways, our study showed the one plausible neurocognitive mechanism of procrastination: the cortical excitability of the DLPFC produced by active neuromodulation may engage prefrontal regulatory networks to increase task-outcome value, which in turn is associated with reduced procrastination behavior, statistically supporting the theoretical accounts of the TDM (Zhang et al., 2019b). TDM uniquely posits procrastination as contingent on the trade-off between task aversiveness and task-outcome value; our observation that only increased outcome value—not decreased aversiveness—statistically predicted reduced procrastination aligns precisely with TDM’s hypothesis that value-based processes may dominate affective-avoidance processes in driving behavioral change. However, neither TMT (which emphasizes temporal discounting of utility per se) nor the mood repair perspective (which prioritizes short-term affect regulation) explicitly predicts this dissociable pattern. Despite statistically supporting the TDM, we acknowledge that alternative neurocognitive mechanisms could contribute to the observed reductions in procrastination. For instance, repeated exposure to the experience-sampling protocol may have enhanced participants’ awareness of task progress or facilitated feedback-based learning, thereby increasing the subjective value of goal completion independent of DLPFC neuromodulation. Participants in the active group may have learned during the repeated sessions that completing tasks feels internally rewarding, thereby benefiting from a form of ‘learned industriousness’ (Eisenberger, 1992) that persists months later. Similarly, improvements in working memory for task maintenance, attentional allocation to reported goals, or strategic shifts in goal selection across sessions could plausibly mediate the intervention effects. While our double-blind, sham-controlled design and inclusion of daily emotional covariates help mitigate some non-specific confounds, the present study did not incorporate direct measures of these alternative processes. Consequently, we cannot definitively isolate the value-based pathway posited by the TDM from concurrent contributions of attention, learning, or executive functions.

In addition, another contribution of the current study is to provide an effective way to reduce both procrastination willingness and actual procrastination in real-life tasks. As mentioned above, despite the fact that multifarious behavioral interventions and evidence have been massively studied for overcoming procrastination, they have shared a common aim—that is—reducing the intention-action gap (Miao et al., 2024; van Hooft et al., 2005). van Eerde and Klingsieck, 2018 put forward an insightful standpoint established by meta-analytic evidence: procrastination is characterized as an intention-action gap rather than an intention to postpone (van Eerde and Klingsieck, 2018). This notion has been partly supported by showing that cognitive behavior therapy for goal-directed behaviors may outperform other interventions focusing on time management (Rozental et al., 2018). Moreover, the trans-theoretical model of procrastination has shown that the behavioral intervention may be effective in changing one’s motivation to overcome procrastination but not in actual behaviors (Grunschel and Schopenhauer, 2015). Thus, the ms-tDCS protocol employed here may offer a promising avenue for reducing procrastination by attenuating the intention-action gap, though further validation in larger, clinically screened cohorts is warranted. Furthermore, both 2-day-interval long-term effects and the 6-month long-term retention of the effects of ms-tDCS on reducing actual procrastination have been revealed as well. Thus far, the trends in adopting tDCS neuromodulation techniques in many aspects of behavioral therapies have emerged, but concern for a long-lasting effect of single session stimulation has continued (Brunoni et al., 2013; Brunoni et al., 2012). To tackle this concern well, instead of single-session tDCS, the current study adopted multiple-session stimulation to implement neuromodulation on the left DLPFC, which facilitates long-term effects (Au et al., 2017; Tedesco Triccas et al., 2016). Existing neurobiological theories and empirical evidence have demonstrated that multiple-session tDCS stimulation could boost cumulative effects of consolidation for activity-dependent LTP, which is crucial to neurobehavioral learning, and thus produce robust long-term after-effects (Agboada et al., 2020; Au et al., 2017). Intriguingly, the activity-dependent LTP process produced by multiple-session consolidation was found to contribute to long-term cortical plasticity, especially in the DLPFC (Jannati et al., 2023; Siebner and Rothwell, 2003). Thus, the detectable effects at 6 months are consistent with the hypothesis that repeated neuromodulation may induce neuroplastic changes in the DLPFC that support sustained behavioral change. However, we explicitly note that a single follow-up timepoint cannot establish the stability or trajectory of these effects; future studies with multiple longitudinal assessments are required to substantiate claims about long-term retention. On balance, this study provided an effective way to help procrastinators overcome actual procrastination in real-life.

While the use of a multi-session design and the long-term assessment can be considered a strength of the present study, it also has several limitations. Even though various tDCS effects have been demonstrated so far, they also tend to be difficult to replicate and sensitive to not yet fully understood context conditions and interindividual differences, which also applies to transcranial magnetic stimulation (TMS) (Valle et al., 2009). To overcome this shortcoming, it will be necessary to establish an individually tailored tDCS protocol to improve the sensitivity of corresponding interventions (Chew et al., 2015). Thus, future research could further improve the effects of tDCS on reducing procrastination by adopting more individualized tDCS protocols. Another limitation is the lack of real-time functional neuroimaging measures to better monitor the impact of our intervention. In the absence of such measures, we had to rely on behavioral indicators to assess the success of the tDCS training. In addition to technical limitations, a major limitation of the current study is the relatively small sample size (total *N* = 46). While this was determined a priori based on our specific pilot study, small samples inherently bear a higher risk of being influenced by outliers and may overestimate effect sizes compared to large-scale meta-analytic expectations for tDCS. Therefore, these findings warrant caution in generalization and necessitate rigorous replication in larger, adequately powered cohorts. Also, considering the lack of medical screening for psychiatric conditions (e.g., ADHD or depression) in this sample, it remains unclear whether these training effects are domain-specific for procrastination. Notably, we explicitly acknowledge that exploratory Quasi-Bayesian mediation analyses, based on observational measures rather than experimentally manipulated mediators, cannot establish causal pathways and are susceptible to inflated Type 1 error rates, as demonstrated in recent simulation studies. These findings should be interpreted strictly as hypothesis-generating and statistically consistent with the proposed theoretical model, rather than as confirmatory evidence of causal mechanisms. Substantiating the precise neurocognitive pathway will require future studies employing stronger causal designs, such as experimental manipulation of task valuation or longitudinal cross-lagged modeling. Moreover, this study did not collect data for assessing participants’ self-control at either baseline or post-neuromodulation. Accordingly, we explicitly note that self-control was not directly measured or manipulated in this study; the observed associations between DLPFC neuromodulation, task-outcome value, and procrastination are consistent with theoretical models positing a role for top-down regulatory processes, but do not constitute direct evidence that self-control mechanisms were engaged. This limitation precludes definitive conclusions about the unique contribution of self-control-related pathways versus alternative neurocognitive mechanisms. In addition, despite instructing to report valid real-life tasks with high probabilities to procrastinate, we did not measure the task difficulty and consistency across sessions for each participant. Consequently, interpreting the effects of neuromodulation to mitigate procrastination as ‘unique contributions’ warrants caution. Given the absence of cathodal HD-tDCS stimulation as a contrast condition in inference, it also warrants caution that the increased DLPFC excitability may not be the exclusive neural mechanism for procrastination. Finally, we explicitly note that a single 6-month follow-up timepoint cannot definitively establish the stability or trajectory of these effects. Future studies incorporating multiple longitudinal assessments (e.g., 1, 3, 6, and 12 months) are required to substantiate claims about long-term retention and to disentangle the precise contributions of neuroplasticity versus behavioral learning.

In conclusion, this study potentially provides an effective way to reduce both procrastination willingness and actual procrastination behavior by using neuromodulation on the left DLPFC. Furthermore, such effects have been observed for 2-day-interval long-term after-effects and were also found for 6-month long-term retention in part. More importantly, this study identified that the ms-tDCS neuromodulation could decrease task aversiveness and increase task-outcome value, and further demonstrated that the increased task-outcome value could predict decreased procrastination, a relationship that is conceptually consistent with theoretical models of top-down regulation and value-based decision-making. In this vein, the current study enriches our understanding of the neurocognitive mechanism of procrastination by showing the prominent role of increased task-outcome value in reducing procrastination. Also, it may inform the development of theory-driven, neuromodulation-informed strategies for behavioral interventions, pending further validation in diverse populations.

## Materials and methods

**Key resources table:** 

Reagent type (species) or resource	Designation	Source or reference	Identifiers	Additional information
Biological sample (*Homo sapiens*)	Adults with chronic procrastination	This paper		Adults scoring >66 on the General Procrastination Scale recruited from Southwest University; *n* = 1682 screened, 53 enrolled and randomized, and 46 included in the final analysis; see Materials and methods, Participants
Software, algorithm	R (v4.4.1)	R Development Core Team, 2024; https://www.r-project.org	RRID:SCR_001905	Statistical analyses
Software, algorithm	lme4	CRAN; https://cran.r-project.org/package=lme4	RRID:SCR_015654	Linear mixed-effects models
Software, algorithm	lmerTest	CRAN; https://cran.r-project.org/package=lmerTest	RRID:SCR_015656	Satterthwaite’s method for LMM
Software, algorithm	emmeans	CRAN; https://cran.r-project.org/package=emmeans	RRID:SCR_018734	Estimated marginal means and simple effects
Software, algorithm	mediation	Imai et al., 2010; https://cran.r-project.org/package=mediation	RRID:SCR_026984	Quasi-Bayesian causal mediation analysis with MCMC sampling
Software, algorithm	glmmTMB	Brooks et al., 2017; CRAN	RRID:SCR_025512	Beta regression for sensitivity analysis
Software, algorithm	ggplot2 (v3.5.2)	CRAN; https://cran.r-project.org/package=ggplot2	RRID:SCR_014601	Data visualization
Software, algorithm	MATLAB (2021)	MathWorks, Inc; https://www.mathworks.com	RRID:SCR_001622	Data processing
Software, algorithm	GraphPad Prism	GraphPad Software; https://www.graphpad.com	RRID:SCR_002798	Data visualization
Software, algorithm	G*Power	Faul et al., 2007	RRID:SCR_013726	A priori power estimation
Software, algorithm	Experience sampling mobile app	This paper		Custom-developed app for ecological momentary assessment of task aversiveness, outcome value, and task-execution willingness; see Materials and methods, Experimental design and procedure
Other	4 × 1 multichannel HD-tDCS stimulation system	Soterix Medical Inc; https://www.soterixmedical.com		Stimulator with 4 × 1 ring multichannel stimulation adapter (MSA); anodal HD-tDCS, 2.0 mA, 20 min per session, 7 sessions; see Materials and methods, HD-tDCS protocol
Other	High-definition neuronavigation system	ANT Neuro Inc, Germany		Target localization of the left DLPFC (F3); see Materials and methods, HD-tDCS protocol

This study fully adhered to CONSORT reporting guidelines and was originally preregistered in the OSF repository (10.17605/OSF.IO/Y3EDT). However, due to the technical constraint related to OSF account service (see Appendix 1 Methods), this OSF page is no longer accessible. For transparency and best practices of open science, based on the original protocol documentation, a preregistration statement has been reconstructed to clarify a priori hypotheses, sample size determinations, and analysis plans for this study (Appendix 1—table 1).

### Participants

Due to the lack of diagnostic criteria for clinical procrastinators, we recruited a large-scale sample (*n* = 1682) to obtain a stable benchmark distribution. Thus, the procrastinators were captured once their procrastination scores were higher than 66 on the General Procrastination Scale (GPS) (see Figure 2A, B). Following this criterion, a total of 186 participants were included initially, which was in accordance with empirical evidence (i.e., 10–15% prevalence of procrastination) (Harriott and Ferrari, 1996). Subsequently, the semi-structured interview was performed to screen those suffering from problematic procrastination and volunteering for this study, thereby enrolling 53 participants (see Appendix 1 Methods). Seven participants were eventually excluded from the analyses because they voluntarily dropped out before experimental completion. All the included participants were screened for depression and anxiety symptoms (see Table 1).

A full randomized block design was used to assign participants to both groups (active neuromodulation group, NM; sham-control group, SC) (see Figure 2C). As the pilot study probing into the effect of single-session tDCS stimulation to change procrastination willingness indicated (*t* = 2.38, p = 0.02, 95% CI [0.14, 1.49]; Xu et al., 2023), statistical power was predetermined by G*Power at a relatively medium effect size (1 − *β* err prob = 0.80, *f* = 0.25), indicating the total sample size at 34 (17 per group) to reach acceptable power. To account for potential attrition, we determined to recruit 36 participants, at least (see Appendix 1 Methods and Figure 2—figure supplement 1). All the participants reported no history for HD-tDCS or neuromodulation. No significant differences were found between groups for any demographic characteristics (see Table 1). This study and protocol have been fully approved by the Institutional Review Board (IRB) of the School of Psychology, Southwest University (China, IRB200301108). All participants provided written informed consent prior to enrollment. Consent to publish was obtained from all participants. The study was conducted in accordance with the Declaration of Helsinki.

### Measurement

The GPS developed by Lay, 1986 was used to quantify one’s chronic procrastination symptom here (Lay, 1986). This scale was widely adopted in many cross-cultural contexts and has been reported to have good psychometric properties (Klein et al., 2019). There were two additional items that we added for lie detection, including the description of ‘the sky is red’ and ‘I have never taken a shower’. If either one was selected as ‘agree’ by the participant, his/her response would be discarded. Internal reliability of the GPS in the current study was found acceptable (Cronbach’s *α* = 0.890). No significant difference was found between groups for GPS scores (*t* = –1.08, 95% CI: [–4.296 1.283], p = 0.283; Jeffreys–Zellner–Siow Bayesian factor, BF, BF_10_ = 0.455, error = 0.020%).

### Experimental design and procedure

#### Nested cross-sectional longitudinal design

This study used a nested cross-sectional longitudinal design to investigate whether the multiple-session anodal HD-tDCS targeting the left DLPFC could reduce actual procrastination behavior and to probe how this effect manifests. To assess procrastination in daily life, we implemented a 15-day protocol alternating between Neuromodulation Days (Days 2, 4, 6, 8, 10, 12, and 14) and Task Days (Days 1, 3, 5, 7, 9, 11, 13, and 15). On the Neuromodulation days, the 20 min anodal HD-tDCS neuromodulation targeting the left DLPFC was performed for the HD-tDCS active group at intervals of 2 days, while the sham-control group received sham HD-tDCS training. This HD-tDCS training was repeated for a total of seven sessions and lasted 15 days (see Figure 1). Crucially, to capture procrastination in ecologically valid contexts, prior to receiving either active or sham HD-tDCS (administered between 09:00 and 18:00), participants were instructed to specify a real-life task they were personally obligated to complete the following day, with a self-defined deadline strictly constrained to 18:00–24:00 to ensure ≥24 hr between stimulation offset and task deadline, thereby isolating offline after-effects. This task should meet the following three criteria: (1) it should be already assigned in the real-world settings; (2) the deadline should be constrained to 18:00–24:00 (see above); and (3) it should be more likely to induce procrastination. By doing so, more than 300 real-life tasks were collected, spanning academic (e.g., ‘submit a statistics homework assignment’), occupational (e.g., ‘draft and email a project proposal’), administrative (e.g., ‘complete online application for Class C driver’s license’), self-improvement (e.g., ‘practice guitar for ≥30 min’), domestic (e.g., ‘do laundry’), and health-related (e.g., ‘running 2000 m for exercise’). The full task list has been tabulated in Appendix 1. As primary outcomes, all the participants were required to report TEW (Zhang and Feng, 2020; Zhang et al., 2019b) for a real-life task 24 hr post-neuromodulation. Thus, procrastination willingness was quantified as 100-TEW score (see underneath for details). Furthermore, we asked participants to report the actual task completion rate (CR) of the task at the deadline (e.g., participant A finished 90% homework at the deadline and reported this situation to us at the deadline). In this vein, the actual procrastination rate (PR) was quantified as 1 − CR.

On the Task Day, we developed a mobile app to implement the ESM for tracking one’s real-time evaluation of task aversiveness and task-outcome value (see Figure 1). The task aversiveness describes how disagreeable one perceives performing a given real-life task to be, whereas outcome value refers to the subjective benefits of the task outcome brought about by completing the task before the deadline (Zhang and Feng, 2020). As theoretically conceptualized by the TDM of procrastination, the perceived task aversiveness is hyperbolically discounted when approaching the deadline, showing sharply discounting when faring away from the deadline but slowly discounting once nearing the deadline (Zhang and Feng, 2020). Thus, considering this nonlinear dynamics inherent in this hyperbolic discounting, the five recording moments of ESM were selected per task a priori by using a log-spaced temporal sampling scheme (Myerson et al., 2001), with increasing sampling density toward the deadline, such as moments of 10:00 (earliest), 16:00, 18:00, 19:30, and 20:00 (deadline). The five sampling points could meet the statistical prerequisite in the hyperbolic model fitting, requiring ≥4 points (Green and Myerson, 2004). To do so, recording moments of tasks were individually tailored for each task per participant in this ESM procedure. To obviate the confounds of daily emotions in task aversiveness evaluation, we used the averaged scores of PANAS (Terracciano et al., 2003) at 10:00 (morning) and 16:00 (afternoon) as anchoring points to quantify one’s daily emotions by using this ESM app. Before each session of HD-tDCS training, each participant was required to report a real-life task whose deadline is tomorrow. To obtain the long-term effect of HD-tDCS (i.e., the interval between HD-tDCS and task completion is at least 24 hr), the task deadline that participants reported was required to be between 18:00 and 24:00. Once a sampling time was reached, this app would send a digital message to require participants to fill out an online form for data collection.

#### Quantification of covariates of interest

Outcome variables of this study were twofold: one is TEW and another is PR. TEW is used to evaluate one’s subjective inclination to avoid procrastination (Zhang and Feng, 2020). In this vein, we used a 100-point scale to require participants to report their TEW (0 for ‘I will definitely procrastinate this task’ and 100 for ‘I will take action to complete this task immediately’). This metric was recorded 24 hr after neuromodulation to examine its long-term effects. PR is used to quantify the extent to which one task has been procrastinated and was calculated as 1 − CR (task completion rate). Critically, at the precise deadline, the app prompted participants to (1) indicate task completion status (yes/no), and if incomplete, (2) report the percentage completed (1–99%), defined as the Task CR, while simultaneously uploading objective evidence (e.g., screenshots of submitted files, photos of physical outputs, system-generated logs, or app-exported records). If the task was actually completed before the deadline, the CR would be 100% and the PR would be calculated as 0% (1 − CR). PR was recorded at the actual task deadline for each participant. We were also interested in re-investigating their actual procrastination by using PR 6 months after the last neuromodulation to test the long-term after-effect of this neuromodulation.

From what has been mentioned above, task aversiveness and outcome value were considered key factors to explain the effect of neuromodulation on reducing procrastination in the current study. To quantify one’s task aversiveness, participants were required to rate their feelings toward the task by using a 100-point visual analog scale (i.e., How do you feel in the current moment when you need to complete this task before the deadline, with 0 for ‘extremely unpleasant’, 50 for ‘totally neutral’, and 100 for ‘extremely pleasant’). Likewise, participants are also required to rate their outcome value by using a 100-point visual analog scale (i.e., How much do you desire to obtain the incentive outcome of this task, with 0 for ‘extremely weak’, 50 for ‘medium’, and 100 for ‘extremely strong’). As articulated in the temporal decision theoretical model above, the task aversiveness evoked by executing a task was temporally dynamic in a hyperbolic discounting pattern, with sharply discounting in faring away from the deadline but slowly discounting in nearing the deadline (Zhang and Feng, 2020). To quantitatively characterize the task aversiveness with consideration for its dynamics, the model-free area under the curve (AUC) was calculated. Specifically, based on the log-spaced temporal sampling rule, task aversiveness was measured by a 100-point visual analog scale at the five sampling moments. Then, the task aversiveness discounting (*A*) was calculated as 1 − (*A*(*t*)/*A*(earliest)), where *t*(earliest) was the earliest sampling point, serving as the reference for immediate execution. Subsequently, using the GraphPad Prism software (v9, 525), the AUC was computed as the trapezoidal integration between task aversiveness discounting and time across five data points, based on the Myerson algorithm (Myerson et al., 2001). By doing so, a higher AUC reflects stronger temporal discounting of task aversiveness along with nearing the deadline, which means that participants experience a faster decline in subjective aversiveness as execution is delayed, yielding lower effective aversiveness and reduced avoidance behavior. As for the task-outcome value, it was theoretically posited as a relatively stable evaluation of the task (Zhang and Feng, 2020). Therefore, it was quantified by the self-reported 100-point visual analog scale after neuromodulation at least 12 hr later, to ensure no online effect.

#### HD-tDCS protocol

The HD-tDCS suit (stimulator and 4 × 1 multichannel stimulation adapter, MSA) that this study used was produced by Soterix Medical Inc, and has been widely verified safe, effective, and reliable for public (Villamar et al., 2013). Based on advanced properties of 4 × 1 MSA, the targeted areas for current flow can be constrained within 2.5 cm^2^ (Villamar et al., 2013).

To position electrodes into targeted areas (left DLPFC), the 10–20 international system for EEG was initially used to mark potential nodes, and determined Cz as the reference point. There is compelling evidence to claim that the left F3 could be used as the target node for modulating the left DLPFC (Seibt et al., 2015; Tsukuda et al., 2025). In this vein, the central anodal electrode was determined onto F3, and four return electrodes surrounded the central electrode at outside of 7.5 cm, including F5, AF3, FC3, and F1 (see Figure 2E). Ramp-up and ramp-down durations were set to 30 s. To further locate the targeted areas, the high-definition neuronavigation system (ANT Neuro Inc, Welbergweg, Germany) was performed. Results indicated the accurate position that we pre-determined by showing a high overlap probability over the left DLPFC (MNI Coordinate: −51 40 18, 94.33% overlapping probability) (see Appendix 1 Methods and Figure 2E). In addition, the coordinates of this targeted area were retrieved from the Brede Database (http://neuro.imm.dtu.dk/services/), and showed highly pertinent functions related to DLPFC.

Before stimulation, participants were informed to clean the scalp to reduce resistance. Then, cotton swabs were used to separate hair until the scalp surface became visible. Subsequently, the electrically conductive gel (about 1.5 ml) was introduced into the plastic casing facilitating constraint of current flow. Next, the Ag/AgCl sintered ring electrodes were placed onto the plastic casing and covered with a cap to lock them in the right positions. To reduce discomfort, the electrode cables were taped elsewhere. Further, the above processes would be re-adjusted if any electrode resistances were found larger than 1.5 units (Villamar et al., 2013). Once all the processes had been completed, the stimulator would be launched.

Participants in the HD-tDCS training group underwent constant electric current of 2.0 mA targeting the left DLPFC for 20 min. Results from the simulation of electric density showed a peak current of ~0.5 mA/cm^2^ at the central electrode and of ~0.125 mA/cm^2^ at the four return electrodes, thereby indicating the safety and effectiveness. As for the sham-controlled group, the stimulator would deliver current flow with 2.0 mA during the first and last 30 s to elicit a sense of electric stimulation for blinding of them. To obtain the pure offline effect, these measures for TEW, task aversiveness, and outcome value were conducted after stimulation at least 12 hr (Bikson et al., 2016).

#### Statistics

All the statistics were implemented by *R* (https://www.rstudio.com/) and *R*-dependent packages.

To clarify whether multiple-session HD-tDCS neuromodulation can reduce procrastination, the general linear mixed-effects model (LMM) was constructed for subjective procrastination willingness (i.e., self-reported visual analog scores) and actual procrastination behavior (i.e., real-world task-CR before the deadline). Here, sex, age, and socioeconomic status (SES) were modeled as covariates of no interest. As the National Bureau of Statistics (China) issued (https://www.stats.gov.cn/sj/tjbz/gjtjbz/), on the basis of per capita annual household income, the SES was divided into seven hierarchical tiers from 1 (poor) to 7 (rich). To obviate subjective rating bias stemming from individual daily mood, we separately measured participants’ daily emotional fluctuation at 10:00 and 16:00 using a self-rating visual analog item (i.e., ‘How do you feel today?’, 0 for ‘completely uncomfortable’, and 100 for ‘definitely happy’). By doing so, the averaged score of those self-rating emotions at the two time points was modeled into the LMM as a covariate of no interest. Given the risks of convergence failure with the two correlated random-effects structure (i.e., treatment days and self-reported emotions), we hypothesized that the random effects are independent, leading to model simplification. Consistent with this assumption, model comparisons favored the simplified independent structure over the full correlated structure for both outcomes. For the actual procrastination model, the simplified model showed lower AIC (∆ = –1.0) and BIC (∆ = –11.8), with a non-significant likelihood ratio test (*χ*^2^(3) = 5.04, p = 0.17). For the TEW model, the simplified model was also preferred (∆AIC = –5.5, ∆BIC = –16.4; LRT: *χ*^2^(3) = 0.51, p = 0.91). This analysis was implemented using the ‘lme4’ and ‘lmerTest’ packages. Employing the ‘emmeans’ package, simple effects were also tested at baseline and post-last-intervention using Tukey-adjusted pairwise comparisons of estimated marginal means from the full LMM, controlling for covariates and random-effects structure. To validate statistical robustness, instead of continuous outcomes for parametric tests, we also conducted a between-group comparison for the number of tasks that procrastination emerges by using the nonparametric *x*^2^ test with *φ* correction or Fisher exact test. Regarding the 6-month follow-up investigation, this LMM was also built to examine the long-term after-effect of neuromodulation on reducing actual procrastination. To examine whether our findings were sensitive to the distributional assumptions of the dependent variables, we re-analyzed the main models using an alternative distributional specification. Given that both PRs (ranging from 0% to 100%) and TEW (measured on a 0–100 visual analog scale) are bounded continuous outcomes, and that PRs frequently reached the lower boundary (0%) in the present study, a Beta regression model with a logit link function was employed for a sensitivity analysis.

To ascertain the neurocognitive mechanism of tDCS in reducing procrastination, the Quasi-Bayesian mediation analysis was used to model the association between the effects of tDCS, task aversiveness/outcome and decreased procrastination. To build upon this model, the tDCS treatments were inputted as independent variables, and the task aversiveness/outcomes were modeled as mediating variables by using the ‘Mediation’ package (https://cran.r-project.org/web/packages/mediation/) (Imai et al., 2010). We estimated these pathway effects (i.e., average mediation effects, *δ*; average direct effect, *ζ*; total effects, *ρ*) by using Markov Chain Monte Carlo (MCMC) sampling. To improve the statistical reliability, the sequential ignorability assumption was tested by using sensitivity analysis. Details for the statistical principles and basis could be found elsewhere (Imai et al., 2010). As these mediation analyses are based on observational measures rather than experimentally manipulated mediators, they do not establish causal pathways. Simulation studies have shown that such analyses can suffer from inflated Type 1 error rates. Results should therefore be interpreted as hypothesis-generating and statistically consistent with the proposed theoretical model, rather than as confirmatory evidence of causal mechanisms.

## Data Availability

We report how we determined our sample size, all data exclusions (if any), all manipulations, and all measures in the study, and the study follows JARS (Appelbaum et al., 2018). Data, analysis code, and research materials are available at Science Data Bank (ScienceDB, https://doi.org/10.57760/sciencedb.35140). Data were analyzed using R, version 4.4.1 (R Development Core Team, 2024), and the package ggplot2, version 3.5.2, as well as MATLAB (2021, MathWorks, Inc), and GraphPad Prism. The following dataset was generated: ZhiyiC 2026Data Repository to "Modulating task outcome value to mitigate real-world procrastination via noninvasive brain stimulation"Science Data Bank10.57760/sciencedb.35140PMC1358128742747014

## Additional files

MDAR checklist

## Appendix 1

### Methods

#### Reconstructed preregistration statement

This study has been originally preregistered in the Open Science Framework (OSF, 10.17605/OSF.IO/Y3EDT) preregistration service on September 2020. However, the creator’s OSF account was suspended and further disabled due to an automated system flag, rendering the original HTML preregistration page inaccessible, including to the authors. To ensure the accessibility and transparency in full adherence with open science best practices, we have reconstructed the preregistration content herein. This reconstructed statement is not post hoc, but reflects the exact hypotheses, sample size, and analysis plan as executed (Appendix 1—table 1).

#### Randomized participants

To randomize participants into neuromodulation and sham-control group, the full randomized block design (FRBD) was conducted here. Furthermore, it could be beneficial from FRBD that the within-group variances can be minimized. In detail, the whole sample would be portioned into *k* blocks, which were manipulated for high homogeneity. For each block, the pairs of participants were then randomly designated to neuromodulation and sham-control group. Thus, adopting FRBD for randomized processes can reap huge fruits to keep within-group homogeneity for each group than do of that full randomized design (King and Eckersley, 2019). The randomized codes came from ‘https://www.random.org/’, which produced random labels by using atmospheric.

#### Estimation for statistical power

To determine adequate statistical power beforehand, we capitalized on the G*Power software to estimate the minimum sample size obtaining the medium effect size (α err prob = 0.05, 1 − *β* err prob = 0.80, *f* = 0.25) by building a repeated measures, within-between, and interaction effect ANOVA model (Faul et al., 2007). In this vein, the medium effect size can be reached once total sample size *n* = 18, noncentrality *λ* = 15.75, critical *F* = 2.1945, numerator Df = 6, and denominator Df = 96. In addition, owing to the repeated measures for each participant, the two-dimensional plot, so-called Power Contours estimation, was used to visualize and calculate the acceptable sample size by corresponding experimental design (Baker et al., 2021). Thus, according to the design this study made, the Power Contours estimation was done up front and indicated the total sample size of this study can attain the adequate statistical power (see Figure 2—figure supplement 1). More detail for how to estimate and plot this map can be found elsewhere (Baker et al., 2021).

#### How to determine one’s socioeconomic status

In line with the Human Connectome Project (HCP) proposed by the National Institutes of Health (NIH), this study also acquired demographic information for each participant, including gender, age, nationality, educational level (years), family’s economic status (poor, modest, middle, and affluent class), family structure (FS), and status of birth (SB). In terms of criteria endorsed by the Food and Agriculture Organization (FAO) of the United Nations (UN) and the financial statement published by the National Bureau of Statistics (NBS) of China in 2019 (http://www.stats.gov.cn/tjsj/), the economic status of the family was rated as poor, modest, middle, and affluent class according to family incomes, with <¥ 45 thousand per year for poor class, ¥ 45–65 thousand per year for modest class, ¥ 65–105 thousand per year for middle class, and > ¥ 105 thousand per year for affluent class.

To determine homogeneity between the HD-tDCS group and sham-controlled group, non-/parametric and Jeffreys–Zellner–Siow Bayesian examinations were adopted to identify significant differences between demographic variables.

#### Semi-structured interview questionnaire

To ensure the high ecological validity, we developed a semi-structured interview questionnaire to test whether participants were eligible for the current study. There were four major profiles to investigate, including evaluation of how they perceived pain from procrastination, whether their social functions were disrupted by procrastination, whether their attitudes were to change procrastination, and whether they accepted our protocol. The outlines of this questionnaire have been provided as below:

1. Please details your identity and investigation proposal; 2. Do you perceive you have procrastination symptoms, and how severe it is do you think; 3. Are you ever assumed you have ‘procrastination disorder’; 4. Do you feel pain due to procrastination and how it impacts your daily life; 5. What types of procrastination are do you think in yourself; 6. Do you think you would suffer from procrastination all the life; 7. Do you want to get rid of procrastination; 8. Have you try to stop procrastination; 9. Do you know neuromodulation technique, such as tDCS or TMS; 10. Do you feel panic or worrisome for medical technique, especially in electric medical technique; 11. Do you feel panic or worrisome for electric current; 12. Do you willing to receive electric medical technique for getting rid of procrastination despite limited skin pains.

#### High-definition neuronavigation system

To ensure the accuracy of targeting location (left DLPFC), this neuronavigation system was used to estimate the location from the skull. This system reported the MNI coordinate of what we predetermined for the first, and would readjust automatically twice to self-verify the estimation accuracy. Subsequently, the brain labels for these coordinates were reported by using the AAL atlas and Brodmann atlas, respectively. Results indicated that the F3 node overlapped the left DLPFC with 94.33% probability, which was cross-checked automatically.

#### Temporal difference-to-difference model

In the current study, in addition to the estimation for the model including all the repeated measures, we still aimed to do a random-sham pre–post test for clarifying whether this HD-tDCS by using a multiple sessions protocol was effective. In this vein, the difference coefficient (Δ) was computed as the difference between the post-test after the last session and the pre-test before the first session, one-by-one for participants. In this vein, the pre–post within-participant differences upon outcome variables can be estimated as the HD-tDCS training effect.

### Results

#### Unexpected event effects

In session 1, a significant unexpected life event effect occurred by the fact that almost all the participants in both groups do not procrastinate (neuromodulation group, NM, procrastination rate (PR): 4.34% (1/23), sham-control group, SC, PR: 8.69% (2/23)). In the follow-up investigation, all the participants reported a strong expectation for this neuromodulation as they received such treatment for the first time. In session 4, the PR was observed to have increased dramatically in both groups (neuromodulation group, NM, PR: 86.95% (20/23), sham-control group, SC, PR: 86.95% (20/23)). In the follow-up investigation, all the participants reported that taking action immediately to complete tasks was hampered significantly due to the weekend effect (Ryan et al., 2010; Stone et al., 2012). In this vein, data for these sessions were excluded from the analysis. Here, the follow-up investigation required participants to report whether their performance in completing tasks was influenced by additional effects. If this was the case, they should report what unexpected events occurred here. The *x*^2^ test was used to examine whether those reported unexpected events confounding task execution posed significant differences between groups across all the sessions (see Appendix 1—table 2).

#### Robustness check for interaction effects

To examine whether Group*Neuromodulation_Session interactions are statistically robust, we reanalyzed interaction effects by removing data from sessions #6, #7, and both, respectively, in which those sessions showed extraordinarily high effectiveness of neuromodulation (i.e., outliers). The findings derived from those nested models showed that all the interaction effects were retained, indicating a high statistical robustness (Appendix 1—Tables 3 and 4).

#### Results of linear probability panel model

Given the panel data pattern (7 longitudinal sessions × task-execution willingness), this study has drawn upon the linear probability panel model (PLM) fitting the task aversiveness and outcome value to task-execution willingness. Specifically, the Fisher’s Augmented Dickey–Fuller Test (ADF) tests were performed to examine whether these data are suitable for this model. Results indicated the stationary properties for all the variables (task aversiveness, DF = –5.78, p < 0.05; outcome value, DF = –5.83, p < 0.05; task-execution willingness, DF = –5.83, p < 0.01). Further, the findings derived from Breusch–Godfrey/Wooldridge test maintained the decision to reject the stability of pooled model (*x*^2^ = 22.75, p < 0.0001). Also, the Hausman test was done for determining what types of models would be accepted. Results indicated that the random effect model was unstable (*x*^2^ = 12.92, p < 0.0015). Thus, the fixed effect model controlling both time and individual variants was adopted as the final one.

#### Results of LMM adjusted for baseline

To minimize false-positive risks, rather than taking time and group as predictors meantime, we reanalyzed these data by remodeling post-neuromodulation procrastination as dependent and remodeling pre-neuromodulation procrastination, group, and other covariates as predictors. Results showed the significantly predictive of pre-neuromodulation procrastination willingness (*β* = 24.48, SE = 6.24, p = 0.0018) and procrastination rate (PR, *β* = 30.66, SE = 8.48, p = 0.0015) to post-neuromodulation ones. Moreover, for those covariates of interest on cognitive mechanisms, these findings are validated as well, showing statistically significant predictions from pre-neuromodulation cognitive processes to post-neuromodulation ones (task aversiveness, *β* = 0.58, SE = 0.23, p = 0.018; outcome value, *β* = 22.86, SE = 5.11, p = 0.0005). This replicated effect has been observed in predicting post-neuromodulation PR by pre-neuromodulation one for 6 months (*β* = 34.79, SE = 11.1, p = 0.005). Taken together, using stringent statistical constraints to adjust baseline, we revealed the same findings compared to traditional LMM.

#### Results of sensitivity analysis to LMM

To assess whether the primary findings were sensitive to the Gaussian distribution assumption, we re-analyzed the main models using Beta regression with a logit link function, which is appropriate for bounded continuous outcomes. Models were fitted using the glmmTMB package (Brooks et al., 2017) in R, retaining the same fixed and random effects structure as the primary models: (1+day_c+Emotions_c || SubjectID).

The results of the Beta regression models corroborated the primary findings. For procrastination rate, the Group × day_c interaction was significant (*b* = –0.182, SE = 0.091, *z* = –2.01, p = 0.045). For task-execution willingness, the Group × day_c interaction was also significant (*b* = –0.375, SE = 0.086, *z* = –4.35, p < 0.001). The main effects of Group and day_c remained significant across both outcomes (all p < 0.001). Complete results are presented in Appendix 1—table 5.

#### Results of sensitivity analysis to mediation model

To examine the robustness and specificity of this mediation model, we conducted several sensitive analyses. Thus, we attempted to re-do this mediation model by replacing the sampling method from bootstrap-based bias-corrected and accelerated (BCa) intervals to ‘bca’ sampling at 5000 simulations. Results demonstrated the robustness of this model by showing the same findings (see Appendix 1—Tables 6 and 7).

Further, we inputted the age and gender as outcome variables into this model for testing whether this mediation model is specific to predict decreased procrastination. As hypothesized, no significant effects were found to predict task aversiveness by using this model, and thus supported the specificity of these findings (see Appendix 1—table 8).

**Appendix 1—table 1.** Reconstructed preregistration statement table.

Question	Hypothesis	Sampling plan (e.g., power analysis)	Analysis plan	Interpretation given to different outcomes
Whether the multi-session HD-tDCS over the left DLPFC could reduce real-world procrastination?	This HD-tDCS neuromodulation could increasingly mitigate real-world procrastination	We capitalize on the G*Power software to estimate the minimum sample size obtaining the medium effect size (α err prob = 0.05, 1 *− β* err prob = 0.80, *f* = 0.25) by building a repeated measures, within-between, and interaction effect ANOVA model (Faul et al., 2007). In this vein, the medium effect size can be reached once total sample size *n* = 18, noncentrality *λ* = 15.75, critical *F* = 2.1945, numerator Df = 6, and denominator Df = 96. In addition, owing to the repeated measures for each participant, the two-dimensional plot, so-called Power Contours estimation, would be used to visualize and calculate the acceptable sample size by corresponding experimental design (Baker et al., 2021)	(a) The generalized mixed-effect linear model (GLMM) would be constructed using 2 (active vs. sham) × 2 (before first neuromodulation vs. after last neuromodulation) full factorial design for procrastination willingness (i.e., self-reported scores) and actual procrastination rate (1 − self-reported task completion rate). (b) Markov Chain Monte Carlo Generalized linear mixed effects model (MCMCglmm) would be built to re-estimate results that probed above for validation. (c) Temporal difference-to-difference model would be used to obtain the difference coefficient (Δ) individually, and independent *t*-tests with Jeffreys–Zellner–Siow Bayesian examinations for between-group differences upon procrastination willingness (i.e., self-reported scores) and actual procrastination rate (1 − self-reported task completion rate) would be conducted. (d) Between-group comparison for the counts of reporting task procrastination across participants are conducted by *x*^2^ test with *φ* correction or Fisher exact test would be carried out. (e) The joint model of longitudinal and survival data (JM-LAD), in conjunction with machine learning algorithm, was adopted to capture multi-session effects individually.	-
Whether the multi-session HD-tDCS over the left DLPFC could reduce task aversiveness and increase outcome value?	Multi-session HD-tDCS could decrease task aversiveness and increase outcome values meanwhile	See above	The generalized mixed-effect linear model (GLMM) would be constructed using 2 (active vs. sham) × 2 (before first neuromodulation vs. after last neuromodulation) full factorial design for task aversiveness (i.e., AUC of five task time points) and outcome value (i.e., AUC of five task time points).	-
Why multi-session HD-tDCS could ameliorate procrastination willingness and real-world procrastination behavior?	(a) Multi-session HD-tDCS could increase task-execution willingness and decrease real-world procrastination rate by undermining task aversiveness. (b) Multi-session HD-tDCS could increase task-execution willingness and decrease real-world procrastination rate by increasing outcome value.	No suitable method to make this sample size estimation	(a) Generalized linear model to correlate Δ _Task aversiveness_ and Δ _Outcome value_ to Δ _Procrastination willingness_. (b) Generalized linear model to correlate Δ _Task aversiveness_ and Δ _Outcome value_ to Δ _Real-worldProcrastination_. (c) Quasi-Bayesian causal mediation analysis to model the task aversiveness as the mediator for interpreting the association of HD-tDCS intervention to changes of procrastination willingness and real-world procrastination. (d) Quasi-Bayesian causal mediation analysis to model the outcome value as the mediator for interpreting the association of HD-tDCS intervention to changes of procrastination willingness and real-world procrastination.	-
Does this multi-session HD-tDCS neuromodulation have a lasting-effect?	This effect can be tested in the 6-month follow-up		(a) The repeated-measure ANOVA model would be built to test real-world procrastination rates across three time points (pre-, post-, and 6-month follow-up test). (b) Jeffreys–Zellner–Siow Bayesian factor model would be used to validate the above results derived from repeated-measure ANOVA.	-

**Appendix 1—table 2.** The number of reporting unexpected event effects in both groups across all sessions. Here, we conducted a post-neuromodulation investigation to require participants to report whether their performance for completing tasks was influenced by additional impacts outside normal conditions, such as ‘get the flu’, ‘get a fever’, ‘mandatory assignment for other tasks’, and ‘unexpected emergency events’. If in this case, they should report what unexpected events occur to uncontrollably disrupt task execution. In the S1, all the 44 participants reported expected effects from the neuromodulation. In the S1, one participant reported having the flu. In the S3, both participants in the NM group reported a mandatory meeting assignment, while one participant in the SC reported a bicycle accident and an additional two reported mandatory meeting assignments. In the S4, all the 39 participants reported that their task performances were fully disrupted by the weekend. S5–S7 reported the similar additional AE mentioned previously.

	S0	S1	S2	S3	S4	S5	S6	S7
NM	0/23	21/23	1/23	2/23	20/23	0/23	3/23	5/23
SC	0/23	23/23	0/23	3/23	19/23	1/23	0/23	3/23
*x* ^2^	-	.023	-	.200	.026	-	-	.500

**Appendix 1—table 3.** LMM to robustness check with outcome as subjective procrastination willingness.

Model	*β* (group*treatment_day)	Std. error (SE)	*T* value	p value
Full model	–7.78	1.79	–4.35	<0.001
Removing session #7	–9.78	2.25	–4.34	<0.001
Removing session #6	–7.27	1.83	–3.98	<0.001
Removing sessions #6 and #7 both	–10.02	3.38	–2.96	0.005

**Appendix 1—table 4.** LMM to robustness check with outcome as real-world procrastination rates.

Model	*β* (group*treatment_day)	Std. error (SE)	*T* value	p value
Full model	–7.38	2.45	–3.02	0.004
Removing session #7	–10.34	3.16	–3.27	0.002
Removing session #6	–6.55	2.53	–2.59	0.011
Removing sessions #6 and #7 both	–11.07	4.36	–2.54	0.013

**Appendix 1—table 5.** Sensitivity analysis results using beta regression.

DV	Effect	*b*	SE	*z*	p	Consistency with Gaussian model
Actual procrastination rate	Group	–0.760	0.202	–3.77	<0.001	Yes (p < 0.001)
	Treatment Day	0.225	0.064	3.50	<0.001	Yes (p < 0.001)
	Group × Day interaction	–0.182	0.091	–2.01	0.045	Yes (p = 0.004)
Task-execution willingness	Group	–1.081	0.282	–3.83	<0.001	Yes (p < 0.001)
	Treatment Day	0.446	0.064	7.00	<0.001	Yes (p < 0.001)
	Group × Day interaction	–0.375	0.086	–4.35	<0.001	Yes (p < 0.001)

**Appendix 1—table 6.** Summary for mediation model in predicting task-execution willingness by using treatment (active ms-tDCS vs. sham) from mediated effect of increased task outcome.

	Estimate	95% lower	95% upper	p value
ACME	21.95***	10.67	34.90	0.0001
ADE	11.14	–2.20	25.10	0.10
Total effect	33.10***	19.00	47.80	0.0001

*p < 0.05; **p < 0.01; ***p < 0.001.

**Appendix 1—table 7.** Summary for mediation model in predicting actual procrastination by using treatment (active ms-tDCS vs. sham) from mediated effect of increased task outcome.

	Estimate	95% lower	95% upper	p value
ACME	30.91**	11.93	49.63	0.002
ADE	2.77	–7.12	13.38	0.61
Total effect	33.68**	18.10	49.74	0.002

*p < 0.05; **p < 0.01; ***p < 0.001.

**Appendix 1—table 8.** Summary for mediation model in predicting task aversiveness by using treatment (active ms-tDCS vs. sham) from mediated effect of increased task outcome.

	Estimate	95% lower	95% upper	p value
ACME	0	0.00	0.00	1.000
ADE	25.09**	8.29	42.00	0.0036
Total effect	25.09**	8.29	42.00	0.0036

*p < 0.05; **p < 0.01; ***p < 0.001.
