# Supplementary material for: Modulating task-outcome value to mitigate real-world procrastination via noninvasive brain stimulation
Source: eLife. 2026 Sep 16;14:RP108241. doi: 10.7554/eLife.108241 (PMC13581287; doi:10.7554/eLife.108241)
Supplement: Peer review history

# Editor report: eLife Assessment

**DOI:** 10.7554/eLife.108241.5.sa0

**Authors:** Kate M Wassum

**Affiliations:** University of California, Los Angeles United States

**Keywords:** Solid, Valuable

This **valuable** cross-sectional longitudinal study leverages high-definition transcranial direct current stimulation to the left dorsolateral prefrontal cortex to examine its effect on procrastination behavior over an extended time span. The cross-sectional longitudinal study a testing of competing models provided **solid** evidence for how stimulating DLPFC impacts real-world procrastination behavior. Whether these results generalize to a larger population will be a significant future direction. This work will be of interest to those interested in cortical function, procrastination, and related states.

# Referee report: Reviewer #1 (Public review)

**DOI:** 10.7554/eLife.108241.5.sa1

Summary:

The authors report the results of a tDCS brain stimulation study (verum vs sham stimulation of left DLPFC; between-subjects) in 46 participants, using an intense stimulation protocol over 2 weeks, combined with an experience-sampling approach, plus follow-up measures after 6 months.

Strengths:

The authors are studying a relevant and interesting research question using an intriguing design, following participants quite intensely over time and even at a follow-up time point. The use of an experience-sampling approach is another strength of the work.

Comments on revised version.

With the last round of revisions, the authors have now addressed my concerns.

# Referee report: Reviewer #4 (Public review)

**DOI:** 10.7554/eLife.108241.5.sa2

Summary:

The current study tested the effects of repeated sessions of tDCS targeting the DLPFC on procrastination behavior. The main outcome is that anodal versus sham DLPFC tDCS reduces procrastination behavior on both a short-term and a long-term scale up to six months after the stimulation sessions.

Strengths:

The current study tests competing models of procrastination with state-of-the-art high-definition transcranial electric stimulation. The study assesses stimulation effects on procrastination on both a short-term and a long-term scale, suggesting that repeated stimulation of the prefrontal cortex reduces procrastination on a time scale of up to six months.

Weaknesses:

The manuscript has already been reviewed and revised before, and it seems that the quality of the manuscript has substantially improved as a result of this revision process. I agree with the other reviewers that one must be cautious with drawing conclusions regarding the cognitive mechanisms underlying this effect, as many different cognitive functions are implemented by the DLPFC.

One aspect of the current results that puzzles me is the strength of the current stimulation effects. Meta-analyses suggest that tDCS shows only small-to-moderate effect sizes (with Cohen's d around 0.5). While the authors report no effect sizes for their statistical models, the small p values, in combination with the unusually small sample size of 18 participants per group, suggests that the effect size must be rather large. Can the authors provide an estimate of the effect size of their stimulation effects? If they are considerably larger than to be expected, could the authors give an explanation for why their stimulation setup is showing much stronger effects than comparable high-definition tDCS studies on cognition or decision making?

Regarding the strengths of the stimulation effects, I moreover found remarkable that the post-test procrastination rate was 100% in all (!) participants in the DLPFC group (figure 3F). I admit that it is hard to trust results that have no individual variation at all. This means that all participants are perfect responders to tDCS, which is again at variance what one typically expects for tDCS (where one usually has many non-responders). Do the authors have an explanation for this?

In any case, I am surprised by the rather small sample size. Due to the small effect sizes for tDCS, it is common to have a minimum of 30 subjects per group in between-subject designs. According to G*Power, a between-subject design with 17 subjects per group could detect only relatively large effect sizes of Cohen's d = 0.99 (alpha = 5%, power = 80%, independent-samples t-test). As explained above, this is far above the effect size that can be expected for tDCS. In addition, small samples bear the risk that results strongly depend on outliers in the data, which might explain the strong effect size observed in the current study. The small sample size should be discussed as a major limitation of the current study and that the results need to be replicated by studies with larger sample sizes. Moreover, to rule out that the results are driven by outlier in the data, the authors should show individual data points in all plots showing empirical data.

Related to this, in the figure showing individual data points (3B/F), I count only around 10 data points per tDCS group for the 18 participants per group. I ask the authors to modify the plot that the data points from all participants can be seen (for example, by adding some noise on the x-axis for participants with the same value on the y axis).

Another surprising aspect of the data is that repeated sessions of tDCS change procrastination behavior up to six months after stimulation. Do the authors think that their tDCS setup leads to such long-lasting neuroplastic changes, and if yes, can they cite prior work where similar dosages of tDCS also showed such long-lasting effects? Or could the results be explained by learning effects, for example because participants in the DLPFC group learned during the repeated tDCS sessions that it feels internally rewarding to finish one's tasks instead of procrastinating them, and they still benefit from this kind of "learned industriousness" 6 months later? In any case, in my view it is important to be more specific about how seven sessions of tDCS can affect behavior half a year later.

Lastly, the link to the data repository works, but I could not inspect the data because I was asked to request access to the data, which I did not do in order to remain anonymous.

# Author comment: Author response

**DOI:** 10.7554/eLife.108241.5.sa3

**Authors:** Zhiyi Chen, Zhilin Ren, Wei Li, ZhenZhen Huo, Zhuanzheng Wang, Ye Liu, Bowen Hu, Wanting Chen, Ting Xu, Leonov Artemiy, Chenyan Zhang, Bernhard Hommel, Tingyong Feng

**Affiliations:** Army Medical University Chongqing China; Army Medical University Chongqing China; Army Medical University Chongqing China; Southwest University Chongqing China; Army Medical University Chongqing China; Army Medical University Chongqing China; Southwest University Chongqing China; Southwest University Chongqing China; University of Electronic Science and Technology of China Chengdu China; Clark University Clark United States; Leiden University Leiden Netherlands; Psychology, Universiteit Leiden Faculteit Sociale Wetenschappen Dresden Germany; Southwest University Chongqing China

The following is the authors’ response to the previous reviews

**Public Reviews:**

**Reviewer #1 (Public review):**

Summary:

The authors report the results of a tDCS brain stimulation study (verum vs sham stimulation of left DLPFC; between-subjects) in 46 participants, using an intense stimulation protocol over 2 weeks, combined with an experience-sampling approach, plus follow-up measures after 6 months.

Strengths:

The authors are studying a relevant and interesting research question using an intriguing design, following participants quite intensely over time and even at a follow-up time point. The use of an experience-sampling approach is another strength of the work.

Comments on revisions:

Overall, I think the authors made many improvements to their manuscript. There are, however, still a number of concerns that first need to be addressed, since it is still not currently possible to fully evaluate the analyses, results, and conclusions presented in the paper. I list these points below:

(1) The authors still use causal language where they must not use causal language. This is true for many places in the manuscript; I am highlighting here just a few places, but the authors nevertheless have to go carefully through the whole manuscript to change these instances.

We sincerely thank the reviewer for this critical and well-taken point. We fully agree that our design (manipulating DLPFC excitability while measuring procrastination, task value, and aversiveness) does not directly measure or manipulate self-control, nor does it rule out alternative neurocognitive mechanisms. Accordingly, we have conducted a comprehensive, line-by-line revision of the entire manuscript to systematically replace causal claims with cautious, hypothesis-consistent language. In response, we have replaced all the wordings that may imply causal inferences, such as impair, cause, boost, by association-consistent phrasing. Furthermore, as you clearly raised below, we explicitly reframed self-control as a hypothesized theoretical construct rather than an empirically verified mediator throughout the whole re-revised manuscript. Please see specific revisions below:

Abstract Section (Page 2, Line 53-59)

“... a mediation analysis indicated a disassociable mechanism: the increase in task outcome value (but not task aversiveness) showed a statistical pattern consistent with accounting for the observed behavioral improvement. In conclusion, these findings are consistent with the hypothesis that enhancing DLPFC function may reduce procrastination by selectively amplifying the valuation of future rewards, not by simply reducing negative feelings about the task.”

Introduction Section (Page 3, Line 83-86)

“... Even worse, chronic procrastination has been consistently associated with poor general health conditions, such as immune system disruption, gastrointestinal disturbance, hypertension and cardiovascular disease (Sirois, 2015; Sirois, 2016).”

Introduction Section (Page 4, Line 143-145)

“Consistent with this framework, the left dorsolateral prefrontal cortex (DLPFC)—a region frequently implicated in value-based decision-making and top-down regulation—has been associated with procrastination. ...”

Introduction Section (Page 4, Line 135-139)

“... Also, given the integrative nature of prefrontal regulatory functions, we hypothesize a third pathway whereby both decreased task aversiveness and increased task-outcome value may jointly contribute to reduced procrastination, potentially reflecting coordinated downstream effects on valuation and affective processing.”

Introduction Section (Page 5, Line 183-186)

“... Thus, this study aims to clarify the brain-behavior association between DLPFC neuromodulation and procrastination, and to test whether observed changes in task valuation and aversiveness are consistent with theoretical models of top-down regulation.”

Results Section (Page 11, Line 534-536)

“... Thus, these findings are consistent with the view that neuromodulation of the left DLPFC is associated with reduced task aversiveness and increased task-outcome value.”

Results Section (Page 11, Line 579-584)

“... In summary, these findings identified a statistical pathway consistent with the theoretical model: neuromodulation of the left DLPFC was associated with increased task-outcome value, which in turn was associated with reduced procrastination.”

Discussion Section (Page 12, Line 617-620)

“On balance, our findings provide evidence consistent with the hypothesis that neuromodulation of the left DLPFC is associated with reduced procrastination, primarily through increasing task-outcome value rather than merely reducing task aversiveness. ...”

Discussion Section (Page 13, Line 664-669)

“... Building on this foundation, among several theoretical interpretations and cognitive pathways, our study showed the one plausible neurocognitive mechanism of procrastination: the cortical excitability of the DLPFC produced by active neuromodulation may engage prefrontal regulatory networks to increase task outcome value, which in turn is associated with reduced procrastination behavior, statistically supporting the theoretical accounts of temporal decision model (TDM, Zhang et al., 2019).”

Discussion Section (Page 14, Line 755-762)

“... Moreover, this study did not collect data for assessing participants' self-control at either baseline or post-neuromodulation. Accordingly, we explicitly note that self-control was not directly measured or manipulated in this study; the observed associations between DLPFC neuromodulation, task-outcome value, and procrastination are consistent with theoretical models positing a role for top-down regulatory processes, but do not constitute direct evidence that self-control mechanisms were engaged. This limitation precludes definitive conclusions about the unique contribution of self-control-related pathways versus alternative neurocognitive mechanisms.”

Some examples:

(a) In response to my comment (1) in the previous round, where the authors adjusted their text, the authors still use causal language in their last sentence "... procrastination behavior has been observed to *impair* general health..." Unless the cited study truly allowed causal conclusions, the causal language should be removed here as well.

Thank you for pointing out this inappropriate phrasing. As you kindly suggested, we have reworded it as “Even worse, chronic procrastination has been consistently associated with poor general health conditions, such as immune system disruption, gastrointestinal disturbance, hypertension and cardiovascular disease” (Introduction Section, Page 3, Line 83-86).

(b) The authors still make (causal) claims about the involvement of self-control in their observed results. To reiterate from the previous round of revisions: The authors cannot make any strong claims about the role of self-control processes because they do not directly measure self-control nor do they directly manipulate self-control or have a design that would rule out alternative mechanisms other than self-control. Therefore, their claims about self-control have to be toned down. It is laudable that the authors have added a statement towards the end of their discussion about not being able to make strong conclusions about the role of self-control. But the authors need to use similar careful wording not just at the end of the discussion but throughout the manuscript.

We appreciate you reiterating this concern. In the re-revised manuscript, we have thoroughly removed or rewritten all the statements implying causal inferences, and have substantially toned-down claims for the roles of self-control in procrastination reduction from this neuromodulation. Please see instances for what we have replied to the Comment #1.

(i) In the abstract, the authors use the formulation "...conceptualized roles of self-control on procrastination..." -- this wording is still too strong, suggesting that you actually studied self-control.

Thank you for providing this specific instance. This inappropriate sentence has been removed.

(ii) In the introduction (page 4, lines162-169), the way the authors formulate these sentences suggests that they directly measured self-control. Again, the authors need to make it explicit that they are not directly measuring self-control but its hypothesized down-stream consequences on valuations/behavior.

Many thanks. This statement has been removed, and we have reworded it as “... Thus, this study aims to clarify the brain-behavior association between DLPFC neuromodulation and procrastination, and to test whether observed changes in task valuation and aversiveness are consistent with theoretical models of top-down regulation.” (Introduction Section, Page 5, Line 183-186).

(iii) In the discussion, for example, on page 11, lines 555 and following, the authors write: "One major contribution this study has made is to disentangle the neurocognitive mechanism of procrastination by demonstrating that self-control could increase task-outcome value so as to reduce procrastination."

As you kindly instructed, we have rewritten this statement as “One contribution of this study is to provide empirical evidence partially consistent with the temporal decision model (TDM), showing that increased task-outcome value—rather than decreased task aversiveness—was statistically associated with reduced procrastination following DLPFC neuromodulation.” (Introduction Section, Page 12, Line 625-628), which no longer implies any conclusions for the role of self-control in this study.

Again, please be aware that you are NOT demonstrating that self-control does anything, since you only measure procrastination rates, outcome values, and task aversiveness. It is possible that mechanisms other than self-control might be relevant for this. Perhaps neuromodulation directly increases outcome values, without involvement of self-control processes. You simply cannot know that and therefore you cannot make those claims in the form that you are making them. You can write that the observed results are consistent with the idea that neuromodulation might have had an effect on self-control and this in turn might have affected outcome values. But you also need to make it explicit that, to substantiate these claims, you would need more direct evidence that indeed self-control was involved. These more careful formulations would not at all reduce the value of your work, but indeed they would rather demonstrate your carefulness in interpreting the results you obtained.

We sincerely thank the reviewer for this exceptionally clear and constructive guidance. We fully agree that our study design does not measure or manipulate self-control, and therefore we cannot demonstrate that self-control processes are causally involved in the observed effects. As you correctly note, it is entirely possible that neuromodulation directly modulates outcome valuation or engages alternative neurocognitive pathways (e.g., attentional allocation, feedback learning, or affective processing) without invoking self-control mechanisms.

In direct response, as we replied above, we have completely rewritten the whole revised manuscript to remove any assertions that we “identified” a role of self-control. The revised text now explicitly states as follow: (1) our findings merely are consistent with the theoretical hypothesis that DLPFC neuromodulation might engage prefrontal self-regulatory functions, which in turn influence outcome valuation; (2) we explicitly acknowledge that substantiating this specific pathway would require more direct evidence. Rather than single sentence, we have applied this careful, hypothesis-consistent framing systematically across the Abstract, Introduction, Results, and Discussion. As you suggested, these revisions more accurately reflect the interpretative boundaries of our data and demonstrate our commitment to rigorous, transparent scientific reporting. Please see specific cases for this revision above.

(2) I am still puzzled by the power analysis. In the text, you write that a sample size of 18 participants (i.e., 9 per group) would be sufficient to achieve 80% power. I still feel this seems far too optimistic and hard to believe, but that is not my point here. While in the text, you write that you need 18 participants, the G*power output seems to suggest a sample size of 34, not 18. Why this contradiction? Or is it not contradictory? If it is not, then please explain it more fully.

We appreciate you pointing out this critical typo. In the last round of revision, we mean that 18 participants per group are required to achieve at least 80% statistical power, rather than a total sample size, as shown by the GPower software. We are sorry for this critical typo to confuse you. As you correctly pointed out, the GPower indicated that the minimum sample size to reach 80% power is 34 (i.e., 17 per group). Thus, we selected 36 (i.e., 18 per group) participants as minimum sample size in case of potential drop-out. We have thoroughly corrected this typo, and double-checked no such numeric issues:

Methods Section (Page 5, Line 234-237)

“... statistical power was predetermined by G*Power at a relatively medium effect size (1-*β* err prob = 0.80, *f* = 0.25), indicating the total sample size at 34 (17 per group) to reach acceptable power. To account for potential attrition, we determined to recruit 36 participants, at least.”.

(3) I have several comments about the mixed-effects analysis.

First of all, I want to thank the authors for adding more details, things have become much clearer now. However, I still have a few questions and comments related to these analyses:

(a) The variable Emotions was within-subjects, as far as I understood. Accordingly, Emotions should most likely be modelled with random slopes varying over participants (in addition to being modelled as a fixed effect).

We thank you raising this reasonable concern on the mixed-effect linear modeling. Yes, the Emotions reflect daily baseline affect, which is modeled as covariates of no interests to adjust for daily emotional fluctuation (if any). In this vein, this baseline emotion score is included for each participant across all the sessions. Therefore, it should be modeled with random slopes as you assumed indeed.

In response, we have remodeled this mixed-effects analysis by including the daily baseline emotion as random slopes varying over participants. After centering the variables, we estimated the revised models as “Procrastination Rate ~ Group * Treatment day + Age + Gender + SES + Emotions + (1 + Treatment day + Emotions || SubjectID)” and “Task execution willingness ~ Group * Treatment day + Age + Gender + SES + Emotions + (1 + Treatment day + Emotions || SubjectID)”. Notably, as you correctly assumed, fitting this complicated random-effect structure is likely to result in convergence failure, given the limited sample size in the present study. Therefore, we hypothesized the independence among random effects for model simplification. Consistent with this assumption, model comparisons indicated that the simplified models fit better than original ones (Procrastination Rate model, ∆AIC = -1.0, ∆BIC = -11.8, LRT, χ^2^(3) = 5.04, *p* = .17; Task-execution willingness model, ∆AIC = -5.5, ∆BIC = -16.4, LRT, χ^2^(3) = 0.51, *p* = .91).

Taken together, as you kindly suggested, we have rebuilt the mixed-effect models by adding daily baseline emotion as random slopes varying over participants, and have demonstrated the consistent findings with the original one:

Methods Section (Page 8-9, Line 412-419)

“... Given the risks of convergence failure with the two correlated random-effects structure (i.e., treatment days and self-reported emotions), we hypothesized that the random effects are independent, leading to model simplification. Consistent with this assumption, model comparisons favored the simplified independent structure over the full correlated structure for both outcomes. For the actual procrastination model, the simplified model showed lower AIC (∆ = -1.0) and BIC (∆ = -11.8), with a non-significant likelihood ratio test (χ^2^ (3) = 5.04, *p* = .17). For the task-execution willingness model, the simplified model was also preferred (∆AIC = -5.5, ∆BIC = -16.4; LRT: χ^2^ (3) = 0.51, *p* = .91).”

Results Section (Page 9-10, Line 469-489)

“For procrastination willingness, results showed a statistically significant interaction effect between multi-session neuromodulations and groups (*β* = -7.84, SE = 1.80, *t* = -4.36, DF = 45.6, *p* < .001; Fig. 3A). In the post-hoc simple effect analysis, it demonstrated a significantly increased task-execution willingness (i.e., decreased procrastination willingness) after neuromodulation in the active neuromodulation group (NM-before: 35.65 ± 30.21, NM-after: 80.43 ± 19.92, Mean Diff = 41.79, SE = 7.58, DF = 103.4, *t*.ratio = 5.51, *p* < .0001, Tukey correction), but no such effects were identified in the sham control group (SC-before: 37.57 ± 26.46, SC-after: 47.35 ± 30.49, Mean Diff = 2.58, SE = 7.56, DF = 96.8, *t*.ratio = 0.34, *p* = .73, Tukey correction) (Fig. 3B-C). A linear uptrend for task-execution willingness was further observed across multiple sessions in the active NM group, indicating gradually increasing neuromodulation effects (Fig. 3D; *p* < .01, Mann-Kendall test). For actual procrastination behavior, changes to actual procrastination rates across all the sessions have been detailed in the Fig. 3E. Similarly, a statistically significant interaction effect was identified here (*β* = -7.37, SE = 2.40, *t* = -3.02, DF = 46.6, *p* = .004), and the simple effect analysis further revealed decreased actual procrastination rates after ms-tDCS in the active neuromodulation group (NM-before: 56.74 ± 39.10, NM-after: 0.00 ± 0.00, Mean Diff = 44.40, SE = 9.36, DF = 110.0, t.ratio = 4.74, *p* < .0001, Tukey correction), but no such prominent changes found in the sham control group (SC-before: 46.47 ± 40.76, SC-after: 33.35 ± 37.82, Mean Diff = 7.53, SE = 9.28, DF = 102.0, t.ratio = 0.81, *p* = .42, Tukey correction) (Fig. 3F-G).”

(b) The analyses still cannot fully be evaluated as I cannot access the scripts and data. The authors mention that the scripts and data should be available via a link they provide (https://doi.org/10.57760/sciencedb.35140). However, when I try to access these materials via this link, no page opens; it seems the link is dead?

Thank you very much for bringing this case to us. We checked this link and found it to be still active.

To ensure accessibility for your evaluation, we have uploaded scripts and data into this online submission system. Please do let us know if you are still unable to access them. We are glad to send them to you by other available pathways. This link is a private access to you, and the repository would be openly available for other users upon the final publication.

(c) What are the results and conclusions if you do not include the covariates of no interest? I.e., please re-run your main models without age, gender, SES, Emotions.

Thank you for raising this question. As you clearly instructed, we have rerun main models without all those covariates. As shown in the table below, the results for the key predictors of interest (Group, Treatment day, and their interaction) remained largely unchanged in terms of effect size, direction, and statistical significance:

**Author response table 1.** Comparison to statistics derived from model with covariates (i.e., age, gender, SES, Emotions) and without covariates.

DV	Effect	Covariate	beta	SE	t	p	95% CI
Taskexecution Willingness	Group	Included	-22.18	5.66	-3.92	<. 001	[-33.50, -10.86]
	Group	Excluded	-21.57	5.50	-3.92	<. 001	[-32.57, -10.57]
	Treatment day	Included	9.53	1.28	7.47	<. 001	[6.97, 12.09]
	Treatment day	Excluded	9.18	1.31	6.99	<. 001	[6.56, 11.80]
			- 6 -				
Actual procrastinat ion rate	Group×Day interaction	Included	-7.84	1.80	-4.36	<. 001	[-11.44, -4.24]
	Group×Day interaction	Excluded	-7.48	1.86	-4.03	<. 001	[-11.20, -3.76]
	Group	Included	-25.96	6.49	-4.00	<. 001	[-38.94, -12.98]
	Group	Excluded	-24.77	6.19	-4.00	<. 001	[-37.15, -12.39]
	Treatment day	Included	8.92	1.73	5.15	<. 001	[5.46, 12.38]
	Treatment day	Excluded	8.99	1.77	5.09	<. 001	[5.45, 12.53]
	Group×Day interaction	Included	-7.37	2.44	-3.02	. 004	[-12.25, -2.49]
	Group×Day interaction	Excluded	-7.44	2.50	-2.98	. 005	[-12.44, -2.44]

(d) The authors mention that they use GLMMs, which would suggest *generalized* mixed-effects models, but they do not describe what family/distribution they used. Since they mention lmerTest and seem to report F-tests, my guess is that they used Gaussian models. However, both their DVs (procrastination rates and their ratings) are bounded variables and at least procrastination rates hit the lower boundary. That can mean that their analyses suffer from inflated Type 1 and/or Type 2 rates. Therefore, please repeat the analyses with an appropriate generalized mixed-effects model (perhaps a beta regression type of model?).

We are very grateful to you for raising this crucial statistical point. As you correctly pointed out, we used the Gaussian distribution in estimating this model. We are sorry to confuse you due to the absence of reporting family/distribution we used. In the original manuscript, we meant “general” linear mixed-effect model, rather than “generalized” one. As you clearly and correctly raised, procrastination rates and willingness are technically bounded, and that procrastination rates frequently reached the lower boundary (0%) in the present study, which are in high risks to be inflated for Type 1 and/or Type 2 error.

Thus, as you kindly suggested, a beta family distribution with logit function is used to reanalyze those main effects of interest. Results are tabulated in Author response table 2.

**Author response table 2.** 

DV	Effect	b	SE	z	p	Consistency with Gaussian model
Actual procrastination rate	Group	-0.760	0.202	-3.77	< . 001	Yes (p < .001)
	Treatment Day	0.225	0.064	3.50	< . 001	Yes (p < .001)
	Group×Day interaction	-0.182	0.091	-2.01	. 045	Yes (p=.004)
	Group	-1.081	0.282	-3.83	< . 001	Yes (p < .001)
Task-execution Willingness	Treatment Day	0.446	0.064	7.00	< . 001	Yes (p < .001)
	Group×Day interaction	-0.375	0.086	-4.35	< . 001	Yes (p < .001)

These convergent results confirm that the critical main effect (i.e., Group and Treatment day) and their interaction remain statistically significant across distributional specifications, and that our primary conclusions are not artifacts of the Gaussian assumption. Taken them together, as you kindly suggested, we have repeated the analyses with beta regression family distribution, which replicated our main findings, potentially supporting their statistical robustness.

Following your suggestion, we have added those results derived from such sensitivity analyses into the revised manuscript:

Methods Section (Page 9, Line 430--436)

“... To examine whether our findings were sensitive to the distributional assumptions of the dependent variables, we re-analyzed the main models using an alternative distributional specification. Given that both procrastination rates (ranging from 0% to 100%) and task-execution willingness (measured on a 0-100 visual analog scale) are bounded continuous outcomes, and that procrastination rates frequently reached the lower boundary (0%) in the present study, a Beta regression model with a logit link function was employed for a sensitivity analysis.”

Results Section (Page 10, Line 506-512)

“... Furthermore, as a sensitivity analysis, we reran the main LMMs using Beta regression distribution with a logit link function, which is appropriate for the both bounded outcomes mentioned above (i.e., procrastination rate and procrastination willingness). The main effects (i.e., Group and Treatment day) and their interaction remained significant for both procrastination rate and willingness (see SI Results and Tab. S5), confirming that our findings are robust to alternative distributional assumptions.”

(e) When reporting the results of the mixed-effects models, the authors report the regression coefficient, standard error, DFs and p value, but not the actual test statistic. Please add the information about the test statistic and report all degrees of freedom (in case of F tests that would be the degrees of freedom of the test and the residual degrees of freedom).

We truly thank you for this nuanced reminder. As you suggested, we have added actual test statistics, including t-values and all degrees of freedom (DF). Please see specific instances below:

Results Section (Page 9-10, Line 469-489)

“For procrastination willingness, results showed a statistically significant interaction effect between multi-session neuromodulations and groups (*β* = -7.84, SE = 1.80, *t* = -4.36, DF = 45.6, *p* < .001; Fig. 3A). In the post-hoc simple effect analysis, it demonstrated a significantly increased task-execution willingness (i.e., decreased procrastination willingness) after neuromodulation in the active neuromodulation group (NM-before: 35.65 ± 30.21, NM-after: 80.43 ± 19.92, Mean Diff = 41.79, SE = 7.58, DF = 103.4, *t*.ratio = 5.51, *p* < .0001, Tukey correction), but no such effects were identified in the sham control group (SC-before: 37.57 ± 26.46, SC-after: 47.35 ± 30.49, Mean Diff = 2.58, SE = 7.56, DF = 96.8, t.ratio = 0.34, *p* = .73, Tukey correction) (Fig. 3B-C). A linear uptrend for task-execution willingness was further observed across multiple sessions in the active NM group, indicating gradually increasing neuromodulation effects (Fig. 3D; *p* < .01, Mann-Kendall test). For actual procrastination behavior, changes to actual procrastination rates across all the sessions have been detailed in the Fig. 3E. Similarly, a statistically significant interaction effect was identified here (*β* = -7.37, SE = 2.40, *t* = -3.02, DF = 46.6, *p* = .004), and the simple effect analysis further revealed decreased actual procrastination rates after ms-tDCS in the active neuromodulation group (NM-before: 56.74 ± 39.10, NM-after: 0.00 ± 0.00, Mean Diff = 44.40, SE = 9.36, DF = 110.0, t.ratio = 4.74, *p* < .0001, Tukey correction), but no such prominent changes found in the sham control group (SC-before: 46.47 ± 40.76, SC-after: 33.35 ± 37.82, Mean Diff = 7.53, SE = 9.28, DF = 102.0, t.ratio = 0.81, *p* = .42, Tukey correction) (Fig. 3F-G).”

(f) Thank you for adding the analysis where you remove the last two sessions. But currently you present them in the manuscript without explaining/motivating why you do this. Please add this motivation, as otherwise it will be puzzling for the reader why you conduct these analyses.

Thank you for this very practical requirement to clarify the motivation of reanalyzing main models from removing the last two sessions. Please see specific explanation as follow:

Results Section (Page, Line 499-506)

“... To systematically test whether such effects are biased by extreme data points or patterns, we reran the main LMMs by iteratively removing data from the last two sessions, which showed extraordinarily high effectiveness from neuromodulation (e.g., all the participants in the neuromodulation group had no actual procrastination behavior in session #6 and #7). Results showed the significant group*neuromodulation sessions interaction effects across all those nested models (removing session #6, #7 or both, all *p* < .05; see SI Results and Tab. S3-4), potentially indicating a statistical robustness from the data pattern.”

(4) Mediation analysis

In your manuscript, you present some mediation analyses. Please be aware that such mediation analyses cannot establish causality and they suffer from extremely high Type 1 error rates (see, e.g., https://datacolada.org/103). My suggestion would be to completely remove all mediation analyses. However, if you want to keep them, then you need to be extremely careful in how you present the results. You need to explicitly mention that you cannot derive any causal conclusions from them and that simulation studies have shown that such mediation analyses suffer from extremely high Type 1 errors.

We sincerely thank you for this exceptionally important methodological guidance. We fully agree that mediation analyses, especially those based on observational measures rather than experimentally manipulated mediators, cannot establish causal pathways and are susceptible to inflated Type 1 error rates, as rigorously demonstrated in recent simulation studies (https://datacolada.org/103).

As you kindly suggested, please allow us to retain those mediation analyses upon explicitly highlighting that this mediation statistical model cannot generate any causal conclusions. In response, we have systematically replaced all instances of “causal mediation” with “statistical mediation” or “exploratory mediation analysis”, and removed causal verbs (e.g., “depends on”, "drives", "explains") in favor of association-consistent phrasing (e.g., “is statistically mediated”, “aligns with the hypothesis that”) throughout the abstract, introduction, methods, results and discussion sections. Furthermore, in the Discussion section, we explicitly reiterated the limitations of extending this mediation associations to causal conclusions. Please see specific modifications underneath:

Abstract Section (Page 2, Line 52-56)

“... While the intervention is significantly associated with both decreased task aversiveness and increased perceived task outcome value, a mediation analysis indicated a disassociable mechanism: the increase in task outcome value (but not task aversiveness) showed a statistical pattern consistent with accounting for the observed behavioral improvement.”

Methods Section (Page 9, Line 448-453)

“... As these mediation analyses are based on observational measures rather than experimentally manipulated mediators, they do not establish causal pathways. Simulation studies have shown that such analyses can suffer from inflated Type 1 error rates. Results should therefore be interpreted as hypothesis-generating and statistically consistent with the proposed theoretical model, rather than as confirmatory evidence of causal mechanisms.”

Methods Section (Page 9, Line 438-440)

“... the Quasi-Bayesian mediation analysis was used to model the association between the effects of tDCS, task aversiveness/outcome and decreased procrastination.”

Results Section (Page 11, Line 568-573)

“As an exploratory analysis, results indicated that increased task outcome value was associated with changes in the task-execution willingness (δ = 21.73, p < .01; ζ = 11.25, p = .07, ρ = 32.99, p < .01, simulation = 1,000; see Fig. 5A) and real-world procrastination (δ = 30.78, p < .01; ζ = 3.05, p = .52, ρ = 33.81, p < .01, simulation = 1,000; see Fig. 5B), in the context of ms-tDCS neuromodulation. ...”

Results Section (Page 11, Line 582-584)

“... Nevertheless, all mediation findings are now explicitly labeled as “exploratory” and framed as quantifying statistical associations consistent with the TDM pathway, not causal mediation.”

Discussion Section (Page 14, Line 747-755)

“Notably, we explicitly acknowledge that exploratory Quasi-Bayesian mediation analyses, based on observational measures rather than experimentally manipulated mediators, cannot establish causal pathways and are susceptible to inflated Type 1 error rates as demonstrated in recent simulation studies. These findings should be interpreted strictly as hypothesis-generating and statistically consistent with the proposed theoretical model, rather than as confirmatory evidence of causal mechanisms. Substantiating the precise neurocognitive pathway will require future studies employing stronger causal designs, such as experimental manipulation of task valuation or longitudinal cross-lagged modeling. ...”

As an example (but the mediation results are mentioned in several places, for example, also in the abstract): On page 10, lines 501-503: What you can causally conclude is that neuromodulation affects your measured variables (outcome values, procrastination rates, task aversiveness), but you cannot conclude that the effect of neuromodulation on procrastination rates causally operates via outcome values. Thus, please adjust the formulation accordingly. The same applies to the mediation section that follows right afterwards (page 10, lines 505-522).

Thank you for offering those specific instances. As we replied above, they have been revised accordingly:

Results Section (Page 11, Line 557-559)

“... Collectively, these findings provide statistical evidence consistent with the hypothesis that the outcome-value pathway may contribute to procrastination reduction.”

Results Section (Page 11, Line 563-582)

“Increased task outcome value is specifically associated with reduced procrastination in the context of neuromodulation

To explore the potential neurocognitive pathways of procrastination, the Quasi-Bayesian mediation analysis was undertaken, with increased task outcome value as a statistically mediated variable. As an exploratory analysis, results indicated that increased task outcome value was associated with changes in the task-execution willingness (δ = 21.73, p < .01; ζ = 11.25, p = .07, ρ = 32.99, p < .01, simulation = 1,000; see Fig. 5A) and real-world procrastination (δ = 30.75, p < .01; ζ = 3.05, p = .52, ρ = 33.81, p < .01, simulation = 1,000; see Fig. 5B), in the context of ms-tDCS neuromodulation. To ensure the statistical robustness and specificity of these findings, the sensitivity analysis was implemented by changing sampling parameters and outcome variables. By doing so, those findings were validated statistically robust, as shown by replicated observations across bootstrapping sampling subsets (see SI Results and Tab. S6-7). Moreover, the results of the control analysis further validated the specificity of these findings by showing a null statistically mediated effect of this model to predict one’s task aversiveness (see SI Results and Tab. S8). In summary, these findings identified a statistical pathway consistent with the theoretical model: neuromodulation of the left DLPFC was associated with increased task-outcome value, which in turn was associated with reduced procrastination.”

(5) In the introduction, the authors introduce several theoretical procrastination frameworks (TMT, mood repair, TDM). Do the results of the current paper help to decide which framework might be the most appropriate, at least for the authors data set? It might be of interest to address this explicitly.

We do thank the reviewer for this insightful theoretical question. We agree that explicitly positioning our findings within the broader theoretical landscape strengthens the conceptual contribution of our work. Upon careful consideration, we believe that our results provide the strongest empirical support for the TDM over alternative frameworks (TMT, mood repair). TDM uniquely posits procrastination as contingent on the dynamic trade-off between task aversiveness and task-outcome value. In the present study, neuromodulation was identified to be associated with both pathways but only increased outcome value statistically predicted reduced procrastination. This aligns precisely with TDM’s hypothesis that value-based processes may dominate aversiveness-avoidance processes in driving behavioral change. Neither TMT (which emphasizes temporal discounting of utility per se) nor the mood repair perspective (which prioritizes short-term affect regulation) explicitly predicts this dissociable pattern. As you kindly suggested, we have extended the Discussion section to explicitly contextualize our findings into this theoretical landscape (Discussion Section, Page 13, Line 669-687).

(6) The language is sometimes hard to understand and seems in quite some places grammatically incorrect. Thus, I think the paper would profit very much from thorough English proofreading.

We sincerely thank you for this practical suggestion. We fully agree that the original manuscript contained grammatical inaccuracies and awkward phrasing that could hinder readability. In response, we have engaged a professional academic editing service to thoroughly proofread and polish the entire manuscript. All sentences have been revised for grammatical correctness, syntactic clarity, and academic tone, while strictly preserving the original scientific meaning and technical terminology. We believe this language improvements have substantially enhanced the readability and overall quality of the paper.

**Reviewer #2 (Public review):**

Summary:

Chen and colleagues conducted a cross-sectional longitudinal study, administering high-definition transcranial direct stimulation (HD-tDCS) targeting the left DLPFC to examine the effect of HD-tDCS on real-world procrastination behavior. They find that seven sessions of active neuromodulation to the left DLPFC elicited greater modulation of procrastination measures (e.g., task-execution willingness, procrastination rates, task aversiveness, outcome value) relative to sham. They show that HD-tDCS reduces task aversiveness and increases task-execution willingness on real-world tasks as quantified by intensive experience sampling methods, providing causal evidence for the role of DLPFC in modulating contextual features to delaying or completing one's goals.

Strengths:

- This is a well-designed protocol with rigorous administration of high-definition transcranial direct current stimulation across multiple sessions. The intensive experience sampling approach which probes and assesses self-relevant task goals is innovative and aims to address an important question regarding the specific role of DLPFC in modulating specific features of chronic procrastination behavior (e.g., task-execution willingness, task aversiveness).
- The quantification of task aversiveness through AUC metrics is a clever approach to account for the temporal dynamics of task aversiveness, which is notoriously difficult to quantify.

Weaknesses:

- While the findings that neurostimulation reduces procrastination behavior is compelling, there remain several alternative interpretations for these effects. For example, it could be that the task-execution willingness isn't increased per se, but rather that the goal completion becomes more valuable as participants learn from feedback or become more aware of their successful attainment of or failure to complete task goals. It is unclear whether the effects could be driven by improved working memory or attention to the reported tasks (and this limitation is addressed by the authors). In short, it is also difficult to examine the temporal dynamics of how these goals are selected across time.

We sincerely thank you for raising these thoughtful and methodologically important points. We fully agree that the observed reductions in procrastination could reflect multiple neurocognitive pathways beyond the value-based mechanism emphasized in our primary analysis.

In response, we have thoroughly removed claims on the “unique mechanism” of value-based pathways to procrastination reduction, and fully substituted language implying exclusive mediation by “value amplification” with more cautious phrasing (e.g., “statistically consistent with a value-based pathway”; “one plausible mechanism among several processes”). In the revised manuscript, we reiterated that the pattern of results, showing increased outcome value predicting reduced procrastination while decreased aversiveness did not, aligned with the Temporal Decision Model, yet does not rule out concurrent contributions from attention, learning, or executive processes. To clearly bring this interpretative boundary of our primary findings for audiences, we have explicitly warranted such cautions in the Discussion Section. Please see specific modifications underneath:

Discussion Section (Page 13, Line 664-669)

“... Building on this foundation, among several theoretical interpretations and cognitive pathways, our study showed the one plausible neurocognitive mechanism of procrastination: the cortical excitability of the DLPFC produced by active neuromodulation may engage prefrontal regulatory networks to increase task outcome value, which in turn is associated with reduced procrastination behavior, statistically supporting the theoretical accounts of temporal decision model (TDM, Zhang et al., 2019). ”

Discussion Section (Page 13, Line 676-687)

“... Despite statistically supporting the TDM, we acknowledge that alternative neurocognitive mechanisms could contribute to the observed reductions in procrastination. For instance, repeated exposure to the experience-sampling protocol may have enhanced participants’ awareness of task progress or facilitated feedback-based learning, thereby increasing the subjective value of goal completion independent of DLPFC neuromodulation. Similarly, improvements in working memory for task maintenance, attentional allocation to reported goals, or strategic shifts in goal selection across sessions could plausibly mediate the intervention effects. While our double-blind, sham-controlled design and inclusion of daily emotional covariates help mitigate some non-specific confounds, the present study did not incorporate direct measures of these alternative processes. Consequently, we cannot definitively isolate the value-based pathway posited by the TDM from concurrent contributions of attention, learning, or executive functions.”

- It is unclear whether the current evidence support long-retention of this neurostimulation intervention. The study includes one 6-month timepoint after the study to examine the long-term retention of the neural stimulation effect. Future studies that evaluate the long-term effects across multiple time points would strengthen the evidence for the robustness of this intervention.

We genuinely appreciate you for this insightful and methodologically reasonable point. We fully agree that a single 6-month follow-up assessment, while valuable, provides only preliminary evidence for long-term retention, and that multiple follow-up timepoints would substantially strengthen claims about the durability of neuromodulation effects. To carefully address this point, we have rephrased the “long-term retention” as “long-term after-effects” throughout the whole revised manuscript, and overall toned down the claims on the retention effects. Moreover, this limitation has been explicitly elaborated in the Discussion section:

Abstract Section (Page 2, Line 49-50)

“... we assessed the effect of anodal HD-tDCS on real-world procrastination behavior at offline after-effect (2-day interval) and long-term after-effect (6-month follow-up).”

Results Section (Page 12, Line 606-608)

“... Therefore, beyond short-term effects, the benefits of ms-tDCS neuromodulation on reducing procrastination were still detectable at a 6-month follow-up, providing preliminary evidence consistent with long-term after-effects.”

Discussion Section (Page 14, Line 721-725)

“... Thus, the detectable effects at 6 months are consistent with the hypothesis that repeated neuromodulation may induce neuroplastic changes in the DLPFC that support sustained behavioral change. However, we explicitly note that a single follow-up timepoint cannot establish the stability or trajectory of these effects; future studies with multiple longitudinal assessments are required to substantiate claims about long-term retention.”

Discussion Section (Page 15, Line 771-775)

“... Finally, while our 6-month follow-up provides preliminary evidence for sustained effects, the use of a single follow-up timepoint limits our ability to characterize the temporal trajectory of retention. Future studies incorporating multiple follow-up assessments (e.g., 1-month, 3-month, 6-month, 12-month) would strengthen evidence for the robustness and durability of this intervention.”

**Recommendations for the authors:**

**Reviewer #1 (Recommendations for the authors):**

Please see my detailed comments above (6 points; several of them with subpoints a, b, c, etc).

Thank you for listing those specific and helpful recommendations above. Please see our detailed response posed above, point-by-point.
